# A fast in silico model for preoperative risk assessment of paravalvular leakage

**DOI:** 10.1007/s10237-024-01816-8

**Published:** 2024-02-11

**Authors:** Michelle Spanjaards, Finja Borowski, Laura Supp, René Ubachs, Valentina Lavezzo, Olaf van der Sluis

**Affiliations:** 1 Philips Innovation and Strategy, High Tech Campus 34, Eindhoven, The Netherlands; 2 Institute for Implant Technology and Biomaterials e.V., Friedrich-Barnewitz-Str. 4, Rostock-Warnemünde, Germany; 3 Eindhoven University of Technology, Groene Loper 15, Eindhoven, The Netherlands

**Keywords:** paravalvular leakage, Preoperative risk assessment, TAVR, In silico modeling, Computational modeling, Thin-film approximation, Aortic stenosis, Procedure planning

## Abstract

In silico simulations can be used to evaluate and optimize the safety, quality, efficacy and applicability of medical devices. Furthermore, in silico modeling is a powerful tool in therapy planning to optimally tailor treatment for each patient. For this purpose, a workflow to perform fast preoperative risk assessment of paravalvular leakage (PVL) after transcatheter aortic valve replacement (TAVR) is presented in this paper. To this end, a novel, efficient method is introduced to calculate the regurgitant volume in a simplified, but sufficiently accurate manner. A proof of concept of the method is obtained by comparison of the calculated results with results obtained from in vitro experiments. Furthermore, computational fluid dynamics (CFD) simulations are used to validate more complex stenosis scenarios. Comparing the simplified leakage model to CFD simulations reveals its potential for procedure planning and qualitative preoperative risk assessment of PVL. Finally, a 3D device deployment model and the efficient leakage model are combined to showcase the application of the presented leakage model, by studying the effect of stent size and the degree of stenosis on the regurgitant volume. The presented leakage model is also used to visualize the leakage path. To generalize the leakage model to a wide range of clinical applications, further validation on a large cohort of patients is needed to validate the accuracy of the model’s prediction under various patient-specific conditions.

## Introduction

Patients who suffer from severe aortic stenosis, which is defined by the narrowing of the valve opening caused by stiffening of the leaflets due to calcifications that restrict the valve movement, are often treated by implantation of a prosthetic heart valve (David et al. 1988; Peterseim et al. 1999). The implantation of the prosthetic valve can be performed surgically. However, for high-risk patients, transcatheter aortic valve replacement (TAVR) is a more common and safer option (Iung et al. 2003).

The TAVR procedure is a minimally invasive technique in which the prosthetic valve is loaded into a delivery device (catheter) and moved to the calcified aortic valve location via the inguinal artery (Russ et al. 2013). Once the catheter, loaded with the crimped prosthetic valve, is in the correct position, the prosthetic valve is unfolded inside the aortic root, thereby pushing the native leaflets out of the way, and restoring the correct flow of blood to the aorta and the systemic arteries.

However, several challenges are associated with the TAVR procedure. An excessive radial expansion force exerted by the TAVR stent on the aortic wall can lead to tissue damage (Hopf et al. 2017; Finotello et al. 2017) and can increase the risk of permanent pacemaker implantation (Rudolph et al. 2023). Insufficient force may cause device migration and paravalvular leakage (PVL) (Lerakis et al. 2013). The risk of insufficient force increases due to the existence of calcifications on the native leaflets, as these can hinder full deployment of the TAVR stent. The risk of excessive force increases when the stent is too large for the aortic annulus of the patient. The prognosis and postoperative quality of life of the patients are greatly impacted by these complications. Therefore, it is important that the right stent design and size is chosen and tailored to the specific patient during procedure planning.

The recent review article by Huang et al. (2023) showed that in silico modeling is a powerful tool in therapy planning for tailoring the optimal TAVR treatment to each patient. The three most used numerical methods are finite element analysis (FEA), computational fluid dynamics (CFD) and fluid structure interaction (FSI). In particular, FEA is used to study the structural mechanics and the deformation of the stent and the aortic root. CFD is mainly applied to perform a hemodynamic analysis and FSI is used to study the interaction between the aortic wall, stent, artificial valves and the blood. The results from numerical simulations can be used to predict short-term and long-term complications of TAVR.

Bianchi et al. (2019) employed a finite element model describing TAVR deployment, complemented with CFD to study the influence of procedural parameters on post-procedure hemodynamics for patient-specific clinical cases affected by PVL. They showed that numerical simulations are a suitable approach for preoperative risk assessment of PVL.

Auricchio et al. (2014) and Morganti et al. (2016) developed a finite element model to study the effect of different deployment strategies on the deformation of the stent. In their work, they varied the implantation depth and release angle and analyzed the final shape of the stent and the stresses induced in the aortic wall. Incomplete stent deployment or an asymmetrical opening of the artificial leaflets can directly impact the performance of the stent and influence the quality of life for patients after TAVR, whereas high induced stresses in the aortic wall can lead to tissue damage (Wang et al. 2015).

To study the feasibility of TAVR in patient-specific geometries, Capelli et al. (2012) developed a finite element method to model the TAVR procedure. Bosi et al. (2020) developed a finite element model and validated the outcome with clinical data. Their model can be used to perform a risk assessment of PVL.

Another major cause of failure of TAVR is the structural deterioration of the implanted artificial valves. This may induce valvular re-stenosis several years after the implantation. Fumagalli et al. (2023) developed a FSI model to identify fluid dynamics and structural indices to predict the possibility of valvular deterioration to assist clinicians in the intervention design. Their results showed there was a correlation between the leaflets’ structural degeneration and the wall shear stress distribution on the proximal aortic wall. Therefore, the model is a first step toward preoperative risk assessment of TAVR degeneration. Brown et al. (2023) developed a computational FSI model where they combined crimping and deployment simulations modeled using the immersed finite element-difference method. Furthermore, they modeled the device behavior across the cardiac cycle in a patient-specific aortic root geometry.

Kovarovic et al. (2023) developed in vitro patient-specific TAVR 3D-printed replicas to evaluate the hydrodynamic performance. Using high-resolution $\mu$CT scans, they reconstructed in silico FSI models of these replicas to quantify thrombogenicity in the PVL channels. This work shows that thrombotic events are highly dependent on patient-specific flow conditions. Anam et al. (2022) studied the risk of PVL flow-induced thrombogenicity in bicuspid aortic valve patients that underwent TAVR. Results for different devices were compared using patient-specific modeling. Their work showed the relevance of in silico modeling for procedure planning of TAVR.

In most of the FEA deployment models developed in literature (including the models mentioned in this introduction), the stent is modeled using 3D elements, making these methods computationally expensive. The use of different element types to model stent expansion was previously investigated by Hall and Kasper (2006), who recommended beam elements for reasons of stability and efficiency. More recently, beam elements were used to model TAVR by Bosi et al. (2020). In this paper, the beam element approach is adopted to limit computation costs.

As mentioned, an insufficient contact area between the aortic wall and the device after TAVR leads to PVL. Two main approaches are used to study PVL. The first approach is to calculate the leakage area between the stent and the aortic wall at non-contact areas (Bosi et al. 2018; Bosmans et al. 2016; Liu et al. 2021; Borowski et al. 2021). The disadvantage is its binary character in the result in the sense that it predicts whether PVL occurs, or it does not; information on the flow rate or flow path is not provided. The second approach is to directly quantify the PVL flow using CFD (Bianchi et al. 2019; Lavon et al. 2019) or FSI (Luraghi et al. 2019; Basri et al. 2020; Pasta et al. 2020) models. Using this approach, the leakage flow can be quantified. A disadvantage, however, is that CFD and FSI approaches are computationally expensive. In preoperative risk assessments, this leads to a delay in patient-specific procedure planning. For this reason, the main focus of this paper is to present a fast alternative to these high-fidelity models to perform preoperative risk assessment of PVL. In particular, it concerns a simplified and efficient model that is able obtain a qualitative prediction of the risk of PVL that can be used in procedure planning to, for example, select the most suitable stent size. To this end, a device deployment model is combined with an efficient, simplified leakage model (ELM) based on the thin-film approximation to calculate the regurgitant volume and assess the risk of PVL. Furthermore, the fluid flux can also be calculated using this model, making it possible to visualize the leakage path past the stent.

The paper firstly introduces the efficient leakage model in Sect. 2. This is followed by a section on the verification of the model (Sect. 3). Next, a proof-of-concept analysis of the model is presented by comparing the leakage results to in vitro experiments and CFD simulations in Sect. 4. The clinical application of the model is highlighted in Sect. 5, where leakage results are presented for different stent sizes deployed in a synthetic average female and synthetic average male aortic root geometry with different degrees of aortic stenosis. Finally, a discussion and the conclusions are presented in Sects. 6 and 7, respectively.

## Efficient in silico leakage model (ELM)

In this paper, an efficient, simplified leakage model (ELM) is presented that is intended to be used for preoperative risk assessment of PVL. The aim of this model is to provide a fast estimation of PVL risk based on a patient-specific geometry, aiding in procedural planning. To ensure the model is fast, several assumptions are incorporated aimed at minimizing computational cost, without losing the required level of accuracy.

### Problem description

**Fig. 1:**
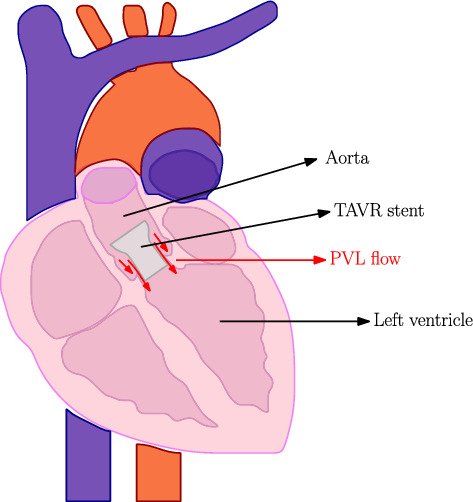
Schematic representation of the TAVR deployed in the aortic root geometry and the paravalvular leakage flow along the stent

As mentioned in Introduction, there are still several risks associated with the TAVR procedure. The focus of this paper is on the risk of PVL, where blood can flow past the stent back into the left ventricle. A schematic representation of the TAVR implanted in the aortic root geometry and the blood flow past the stent is shown in Fig. 1.

### Numerical method

To make the presented model fast and reduce the computational costs for procedure planning, several assumptions are made. Since PVL is of importance during the diastolic phase, the flow can be assumed to be mainly pressure driven. Furthermore, the mean aortic pressure (MAP) is assumed to be constant during the diastolic phase and we neglect turbulence effects. Therefore, the flow is also assumed to be stationary and a Poiseuille flow profile is used to describe the flow in the leakage gap. Additionally, the length scales of the leakage path are assumed to be significantly larger than the thickness of the leakage gap. Therefore, the thin-film approximation is used to model the leakage flow. To this end, the Reynolds equation for pressure driven, stationary, incompressible, viscous Poiseuille flow is solved, based on the thin-film approximation (Shvarts and Yastrebov 2018):

1 $$\begin{aligned} \varvec{\nabla }\cdot \Big [-\frac{h^{3}}{12\mu }\varvec{\nabla } p\Big ] = 0, \quad \quad \quad \quad \text {in}\; \Omega , \end{aligned}$$

where, $\mu$ is the viscosity of blood ($\mu =0.0035\;\text {Pa}\cdot \text {s}$, as given in Nader et al. (2019)), $h=h(x,y,z)$ is the gap size between the stent and the tissue in the fluid domain $\Omega$, and *p* is the fluid pressure.

**Fig. 2:**
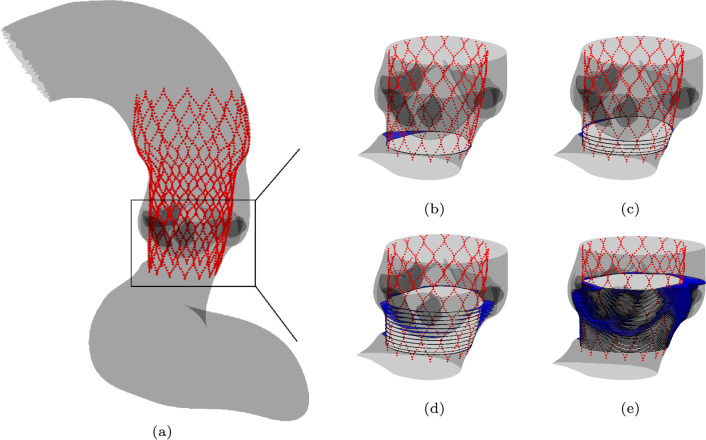
Visualization of the stent (red) deployed in the aorta (gray) (**a**) and a schematic of the construction of the paravalvular gap slices at different heights, where the fluid circumferential area is indicated in blue and the stent circumferential area in white. For visualization purposes, a subset of the total number of slices are plotted (**b**, **c**, **d**). All slices used to construct the gap mesh are plotted in (**e**)

**Fig. 3:**
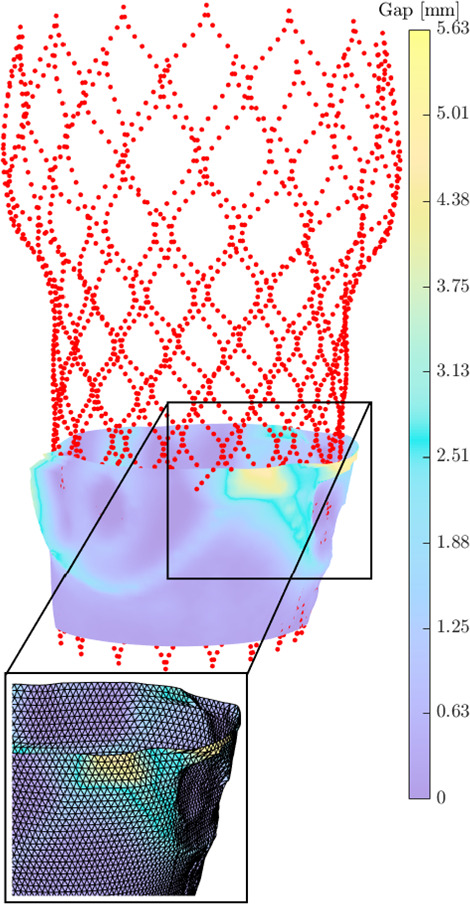
A shell mesh (shown in the magnification inset) is constructed in the radial center of the gap between the aorta and the stent. The colors indicate the gap size *h*

To reconstruct the total leakage volume, first the circumferential shape of the stent and the tissue is obtained at different heights perpendicular to the device’s axial orientation, within the vicinity of the aortic annulus. This is schematically shown in Fig. 2, where a schematic of the construction of the paravalvular gap close to the valves is plotted including the axial slices. Next, a mesh constructed of triangular shell elements, is positioned in the radial center of the gap between the aorta and the stent in the region of interest (close to the valves). This is shown in Fig. 3.

Equation (1) is solved on the shell mesh, where the gap size is given as an additional degree of freedom in the nodes. Using the pressures *p* obtained from solving the Reynolds equation, the fluid flux can be calculated:

2 $$\begin{aligned} \varvec{q} = -\frac{h^{3}}{12\mu }\varvec{\nabla }p, \end{aligned}$$

which can be used to visualize the path along which blood can flow past the stent. At positions in the reconstructed volume where the gap is zero, the flow resistance will be extremely high, leading to dead-end paths. Therefore, dead-end paths and converging paths are accounted for in the ELM.

Equation (1) is discretized and solved using the FE Method in MATLAB.^1^ The weak formulation is obtained by multiplying Eq. (1) with a test function and integrating over the fluid domain using partial integration and applying Gauss’ theorem. The weak form is discretized using linear shape functions and the gap size *h* is calculated in the integration points. From solving the weak form, the regurgitant flow rate is calculated; details are provided in Appendix 1.1. The weak form of Eq. (1) is solved element wise in a local coordinate system in the plane of these shell elements. For more information on the shell element implementation, see Appendix 1.2. Finally, the fluid flux $\varvec{q}$ is obtained and can be plotted to visualize the gradient of flow. More information on the numerical implementation to obtain the fluid flux can be found in Appendix 1.2.

### Input geometry generation

The deployed stent inside the aortic root geometry is needed as input for the ELM, to reconstruct the leakage volume. In this paper, this input is generated using a simplified 3D deployment model. Here, a CoreValve Evolut stent based on the Medtronic CoreValve Evolut is deployed in a synthetic aortic root geometry of an average male and female patient.^2^ CoreValve Evolut stents of different sizes are reconstructed based on micro Computer Tomography ($\mu$CT) images.

The commercial HyperMesh software package,^3^ in combination with the explicit RADIOSS solver is used model the device deployment by calculating the displacements of each component (i.e., stent, aorta and leaflets) based on the applied forces and boundary conditions. An explicit integration scheme is used to solve the equations of motion. A node to surface contact algorithm is used, where, when two components between which contact is defined approach each other, a counteracting external contact force is applied to prevent the components from penetrating. This contact force is then applied as a boundary condition for the next time step. Pre- and post-processing is done using MATLAB and HyperView. More information on the deployment model can be found in Appendix 2.

### Boundary conditions

**Fig. 4:**
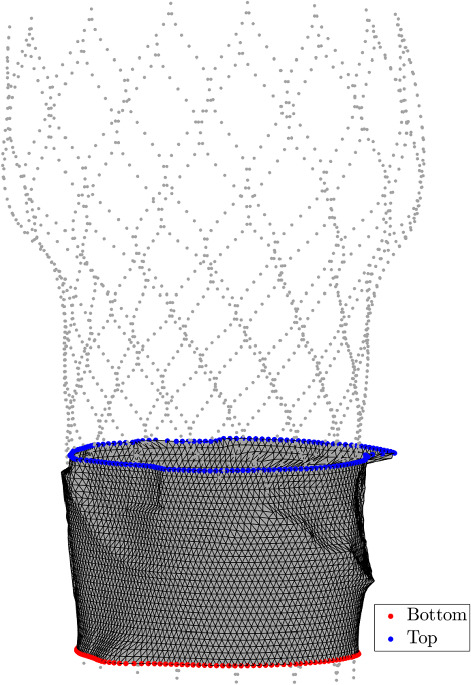
Schematic representation of the essential boundary condition nodes on the shell mesh. On the top (blue) and bottom (red) boundary the pressure is prescribed as an essential boundary condition on the nodes, resulting in a pressure difference over the region of interest for PVL

A pressure gradient, corresponding to the pressure difference over the aortic valve, is prescribed by applying essential boundary conditions in the nodes on the top and the bottom of the shell mesh, as schematically shown in Fig. 4. The mesh shown in this figure is a relatively coarse mesh to enhance its readability. The meshes used in the simulations are more refined.

A characteristic MAP over the valves in healthy adults is $\Delta p = 100$ mmHg (Feher 2012). At the bottom boundary, $p=p_{0}$ is prescribed, while at the top boundary $p=p_{0}+\Delta p_{\textrm{app}}$ is prescribed. Here, $p_{0} = 0$ Pa, and $\Delta p_{\textrm{app}}$ is the applied pressure difference.

**Fig. 5:**
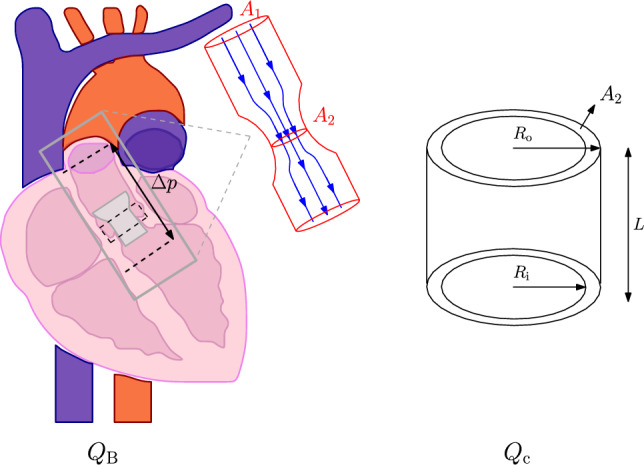
Schematic representation of the resemblance of the PVL problem to ‘flow through an orifice’ and the comparison with the ‘annular die’ approach of the shell mesh. Note: in this figure the annular die is schematically represented with a fixed gap size, whereas in the shell mesh this gap size is variable in axial and tangential direction

The flow through the leakage gap shows resemblance with the flow through an orifice, as is schematically shown in Fig. 5(left). According to Bernoulli, the flow rate in the leakage orifice scales with the ratio of the areas of the main vessel (aorta) and the leakage orifice:

3 $$\begin{aligned} Q_{\text {B}} = C_{\textrm{d}}\sqrt{\frac{2\Delta p}{\rho }}A_{2}\Biggl (1-\biggl (\frac{A_{2}}{A_{1}}\biggl )^{2}\Biggl )^{-\frac{1}{2}}, \end{aligned}$$

where $A_{1}$ is the cross-sectional area of the aorta, $A_{2}$ is the cross-sectional area of the leakage orifice, $\rho$ is the fluid density and $C_{\textrm{d}}$ a coefficient to correct for the kinetic energy losses due to viscous effects and inertia. The value of $C_{\textrm{d}}$ depends on the ratio of the hydraulic diameter of the leakage orifice and the diameter of the aorta and can be estimated from literature (Rouse  1978).

**Fig. 6:**
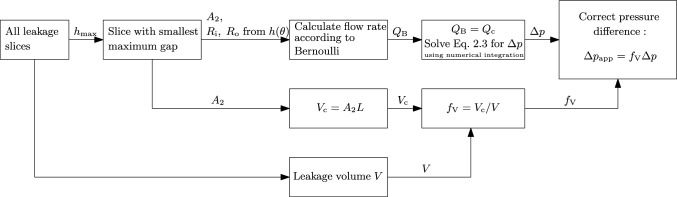
Flowchart representation of the steps taken to obtain $\Delta p_{\textrm{app}}$ from the MAP to make the ‘annular die’ approach of the ELM more consistent with the ‘flow through an orifice’ approach, characteristic for PVL

In the case of a clear leakage path, due to calcifications, the shell mesh used in the ELM approximates a part of an annular die with a variable inner and outer radius over the tangential direction $\theta$ in $[0,2\pi ]$; see Fig. 5(right). Therefore, the pressure difference $\Delta p$ applied over the shell mesh should be scaled to take into account the dependence of the flow rate on the ratio between the cross-sectional area of the aorta and the leakage orifice. This scaling approach makes the ELM more consistent with the ‘flow through an orifice’ problem characteristic for PVL. To this end, the analytical flow rate for an annular die, with an angular variation in the gap, is used. This flow rate can be obtained from the Navier–Stokes equation:

4 $$\begin{aligned} Q_{\text {c}} = \int _{0}^{2\pi }\frac{\Delta p R_{\textrm{o}}^{4}}{16\mu L}\Big [-1 + B^{4}+\frac{\Big (1-B^{2}\Big )^2}{\ln (B^{-1})}\Big ] \textrm{d}\theta , \end{aligned}$$

where $Q_{\text {c}}$ is the flow rate of a fluid with viscosity $\mu$ flowing in an annular die with axial length *L*, $B=R_{\textrm{i}}/R_{\textrm{o}}$ with $R_{\text {i}}$ the inner radius and $R_{\text {o}}$ the outer radius (both dependent on $\theta$) of the die/gap. To obtain the scaled pressure difference, suitable to be applied as a boundary condition over the shell mesh, the steps represented in the flowchart in Fig. 6 are taken, using the leakage slices as represented in Fig. 2e. After construction of the leakage volume, the maximum gap size $h_{\textrm{max}}$ of every slice is saved. The slice that theoretically influences the flow rate the most is picked to perform the scaling. It is assumed that this is the slice with the smallest maximum gap size. The cross-sectional area $A_{2}$ of that slice is calculated and the inner and outer radii ($R_{\textrm{i}}(\theta )$, $R_{\textrm{o}}(\theta )$) for every *n* tangential intervals in the range $\theta$ in $[0,2\pi ]$ of the slice are obtained. These parameters are used to calculate the flow rate according to Bernoulli, using Eq. (3). Equation (4) is rewritten to calculate the pressure difference corresponding to this flow rate. The equation is solved by performing numerical integration, using the trapezoid rule. The obtained pressure difference now corresponds to an annular die of axial length *L* and a cross-sectional shape corresponding to the chosen leakage slice. This means that the volume of this approached annular $V_{\textrm{c}}=A_{2}L$ is not the same as the total leakage volume $V=\sum A_{\textrm{slice}}\Delta l$, with $A_{\textrm{slice}}$ the area of all separate leakage slices and $\Delta l$ the axial distance between the slices. Therefore, the obtained pressure difference is corrected for this difference in volume: $\Delta p_{\textrm{app}} = f_{\textrm{V}}\Delta p$, where $f_{\textrm{V}}=V_{\textrm{c}}/V$.

These steps are implemented in MATLAB. Note that the leakage slice for scaling the pressure difference is only used for the purpose of finding a suitable boundary condition. The flow rate and the fluid flux are calculated by solving Eq. (1) on the complete shell mesh as schematically represented in Fig. 3.

## Verification and validation

The ELM is only valid for small spatial variations in gap size due to the thin-film approximation. Extensive numerical verification on the influence of spatial gradients in the gap *h* on the accuracy of the ELM is performed in Appendix 3.

To validate the shell element implementation of Eq. (1), a simulation was performed on a annular die with an inner radius $R_{\textrm{i}}=10$ mm, an outer radius $R_{\textrm{o}}=11$ mm and length $L=30$ mm. A pressure difference $\Delta p=100$ mmHg $\approx 13.32$ kPa was applied between the top and the bottom of the annular die and the fluid was given a viscosity of $\mu =0.0035\;\text {Pa}\cdot \text {s}$. The analytical solution for the flow rate through the annular die is given by Eq. 4. According to this equation $Q=697.6$ ml/s, whereas a flow rate of $Q=697.2$ ml/s was obtained from the ELM, which corresponds to an error made by the ELM smaller than $1\%$, for the annular die case.

## Proof of concept of the ELM

To give a proof of concept of the ELM, leakage calculations using this model are compared to the results of in vitro experiments and CFD simulations. The in vitro experiments result in a leakage volume with one clear leakage path. To test whether the ELM provides reliable results when there are multiple calcifications involved, simplified aortic root geometries with multiple calcifications are constructed and used as input for the ELM and CFD simulations.

### In vitro model

**Fig. 7:**
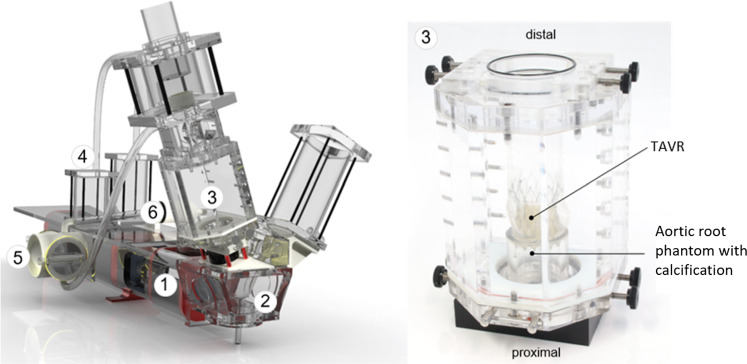
Illustration of the pulse duplicator system (left) with the measurement chamber containing the aortic root model with calcification and the deployed TAVR (right), adapted from Borowski et al. (2023)

In vitro experiments are conducted to investigate the leakage rate past a $\textrm{Portico}^{\textrm{TM}}$^4^ TAVR deployed in a model of a calcified aortic root. Physiological pressure and flow conditions in the aortic root are generated using a commercial pulse duplicator system from ViVitro Labs Inc. (Victoria, Canada), see Fig. 7. The experimental setup was previously described in (Borowski et al. , 2023).

The pulse duplicator system comprises several components, including a digital piston pump (1), a ventricular compressible membrane (2), a measurement chamber with the aortic root phantom (3), a Windkessel to model the compliance of the aortic root and aorta (4), a heat exchanger (5) and a peripheral flow resistance (6). In addition, pressure sensors are attached proximal and distal to the aortic root phantom, as well as a flow sensor located proximal to the TAVR. To provoke an increased leakage rate, a generic calcification model is implemented in the annulus region of the aortic root phantom. The aortic root phantom is made of a two-component elastomer (Sylgard 184 Silicone Elastomer, Dow Chemical Company, Midland, Michigan USA). To ensure better radiopacity of the elastomer model, the silicone is enriched with barium sulfate. This allows for a precise differentiation between the TAVR stent, the aortic root and the lumen when the geometries are reconstructed to be used in the simulations. According to the recommendations of ISO 5840-1:2021 (ISO 2021), a nodule with a radial protrusion of $d_{\textrm{r}} = 2.0\;\textrm{mm}$ and a circumferential extension of $d_{\textrm{c}} = 4.0\;\text {mm}$ into the vessel lumen is applied as a calcification. The calcification nodule is made of a rigid polymer (Young modulus 2.2 GPa) and was glued into the aortic root model.

A mixture of glycerol and saline solution is used as the test fluid. The mixture is adjusted to a viscosity of $\mu =0.0035$ Pa$\cdot$s, which corresponds to the representative viscosity used in the simulations. The pulse duplicator system is used to adjust cardiac parameters such as stroke volume, heart rate, and MAP. Three different parameter sets with different MAP (80, 100, 120 mmHg) are chosen for validation purposes. The remaining cardiac parameters are chosen according to the recommendation of ISO 5840-1:2021, i.e., a heart rate of 70 bpm, a stroke volume of 100 ml, a systolic duration of 35$\%$. The flow sensor is used to measure the leakage rate during the diastolic phase of the cardiac cycle. In addition, the pressure difference proximal to distal to the TAVR during the diastolic phase is measured and used as a boundary condition for the leakage simulations. Note that in the ELM, this pressure difference is scaled, as previously remarked in Fig. 6.

### CFD model

CFD simulations are conducted for comparison to the ELM. Furthermore, it is tested whether the CFD results agree with the experimental results. The CFD simulations include more details of the flow behavior, such as turbulent flow effects. The ELM results are compared to CFD simulations to validate that they give qualitatively the same results.

#### Geometry generation

**Fig. 8:**
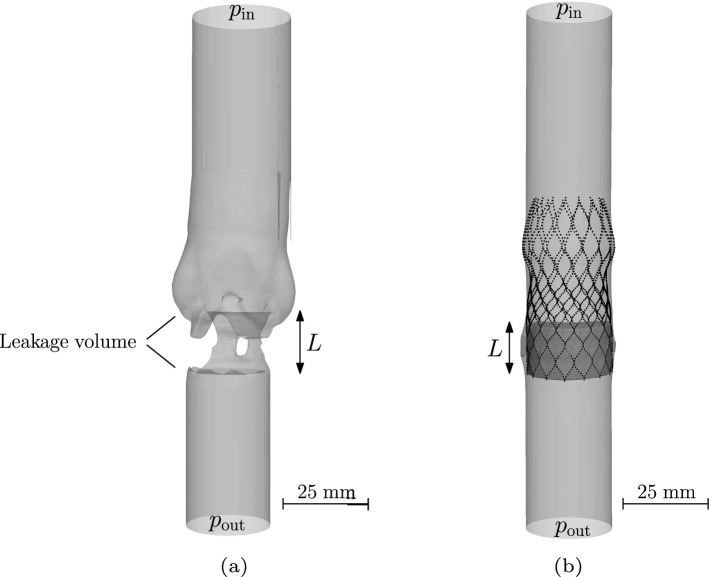
**a** Reconstructed geometry used in the CFD and ELM simulations of the experiments. **b** Representation of the rigid body created from the deformed TAVR. This geometry is used as input for the test cases with multiple calcifications for the ELM and CFD simulations

In order to compare results of the CFD model and the ELM with the in vitro experiments, the geometry of the leakage gap is reconstructed for the simulations using micro computer tomography ($\mu$CT) images. Segmentation of the lumen is performed semi-automatically using MATLAB’s Volume Segmenter app. The reconstructed lumen thus represents the input geometry used for both, the CFD model and the ELM, as represented in Fig. 8a. For the test cases including multiple calcifications (see Sect. 4.4), a simplified geometry is prepared based on the results of TAVR deployment simulations in a hollow cylinder including multiple calcifications. Therefore, a rigid body with a length of $L=15$ mm, representing the skirt and closed leaflets, is created from the deformed TAVR using Blender 3.5.0,^5^ as is schematically shown in Fig. 8b. The computational mesh is generated using the open-source software package OpenFOAM.^6^ The flow calculation was performed with Ansys Fluent.^7^

#### Numerical method

In analogy to the ELM, blood is modeled as a homogeneous, incompressible Newtonian fluid with dynamic viscosity $\mu = 0.0035\;\text {Pa}\cdot \text {s}$ (Nader et al. 2019), and density $\rho = 1060\;\text {kg}/\textrm{m}^{3}$ (Luraghi et al. 2019). The measured volume flow during diastole was nearly constant in the in vitro experiments. Additionally, calculated flow rates for transient and steady-state simulations for the artificial ‘small nodules’ and ‘big nodules’ geometry (both including multiple calcification nodules) were compared and showed to be nearly identical (relative difference of $2\%$), justifying a steady-state assumption of the flow. More information on the transient model and the obtained results can be found in Appendix 4.

To calculate the flow, the Reynolds-averaged Navier–Stokes (RANS) equations are solved, considering the $\kappa$-$\omega$ SST turbulence model. The $\kappa$-$\omega$ SST model was selected due to the presence of free leakage flow jet stream near the tissue below the TAVR.

The magnitude of the mean diastolic pressure difference is defined by setting the pressure at the inlet, while a reference pressure of $p_{0}=0$ Pa is established at the outlet. Additionally, a no-slip condition is applied to all other walls.

A mesh convergence study was conducted. Meshes with up to 12 million elements were used. From an element number of 5 million elements, the change in the leakage rate was sufficiently small ($\le 2\%$). The leakage flow is determined by multiplying the area-averaged velocity perpendicular to the outlet by the outlet area.

### Single calcification

**Fig. 9:**
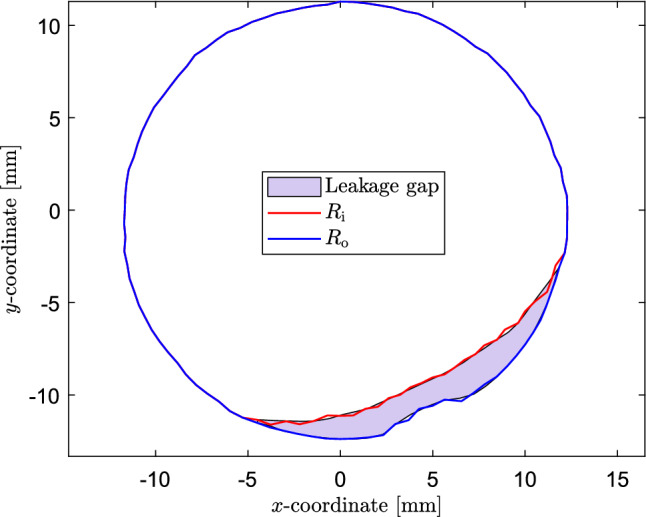
Cross-sectional shape of the gap, used to scale the pressure boundary condition for the ELM (light blue area), and the corresponding estimated ‘concentric cylinder’ shape (red and blue lines) for the performed leakage experiments

Experiments are performed for three different MAPs. The axial slice with the leakage gap picked for scaling the pressure boundary condition for the ELM, as explained in Sect. 2, and the corresponding estimated ‘concentric cylinder’ shape is shown in Fig. 9.

**Fig. 10:**
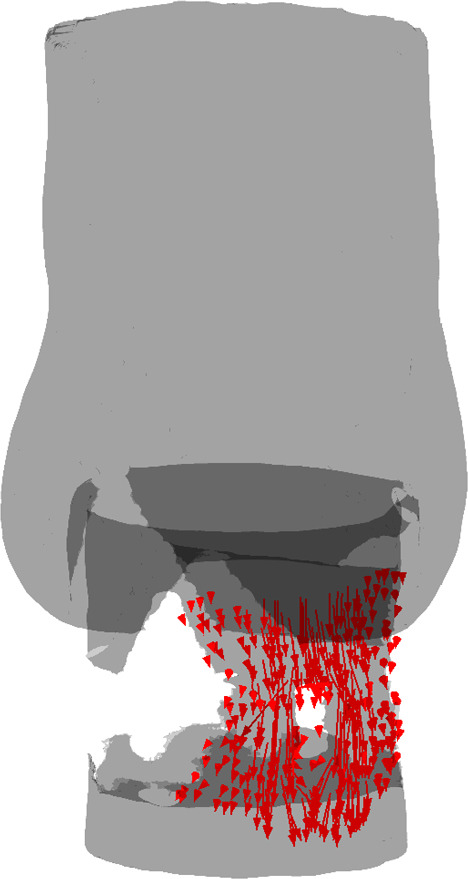
Regurgitant flow for a MAP of 80 mmHg visualized in the fluid segmentation obtained from the experiments

For real patients, the PVL classification of Kappetein et al. (2012) can be used to estimate the risk of postoperative problems. In this work, PVL was classified via the regurgitant volume (RV) as follows: no leakage, mild leakage ($<30$ ml), moderate leakage ($30-59$ ml) and severe leakage ($>60$ ml). The ELM gives a steady-state prediction of the regurgitant flow rate in ml/s. For comparison to the PVL classification of Kappetein et al. (2012), the result has to be converted to a regurgitant volume. Considering a heart rate of 70 bpm and a diastolic duration of $65\%$ (ISO 2021), the duration of the diastolic phase can be calculated to be approximately 0.56 s. This time can be used to convert the flow rates expressed in ml/s to a regurgitant volume. In the remainder of this paper, all flow rates are translated into a regurgitant volume following this reasoning. The experimental and numerical results for the regurgitant volume are shown in Table 1. The regurgitant volumes can be classified as mild leakage for $\textrm{MAP}=80\;\textrm{mmHg}$ and moderate leakage for $\textrm{MAP}=100,\;120\;\textrm{mmHg}$. The fluid flux is visualized in Fig. 10. This figure shows that there is a clear leakage path past the calcification nodule.

**Table 1:** Comparison of experimental results to CFD simulations and the ELM

$\text {MAP (mmHg)}$	$\varvec{\textrm{RV}_{\textrm{exp}}} \text {(ml)}$	$\varvec{\textrm{RV}_{\textrm{CFD}}} \text {(ml)}$	$\varvec{\textrm{RV}_{\textrm{ELM}}} \text {(ml)}$
80	29	27	24
100	31	30	26
120	33	33	28

The maximum error between the experimental results and the ELM equals 18$\%$ for $\textrm{MAP}=80\;\textrm{mmHg}$. The largest error between the experimental results and the CFD simulations equals $5\%$ and the largest error between the ELM and the CFD simulations equals $14\%$. It has to be noted that both models are highly sensitive to the gap size since the flow rate is proportional to $R_{\textrm{o}}^{4}$ and $R_{\textrm{i}}^{4}$ (see Eq. (4)). Hence, the accuracy of the calculated regurgitant volume significantly depends on the quality of the segmentation of the leakage volume and the mesh for both models. The results presented in Table 1 indicate that both models align well with the experimental measurements and with each other.

### Multiple calcifications

**Fig. 11:**
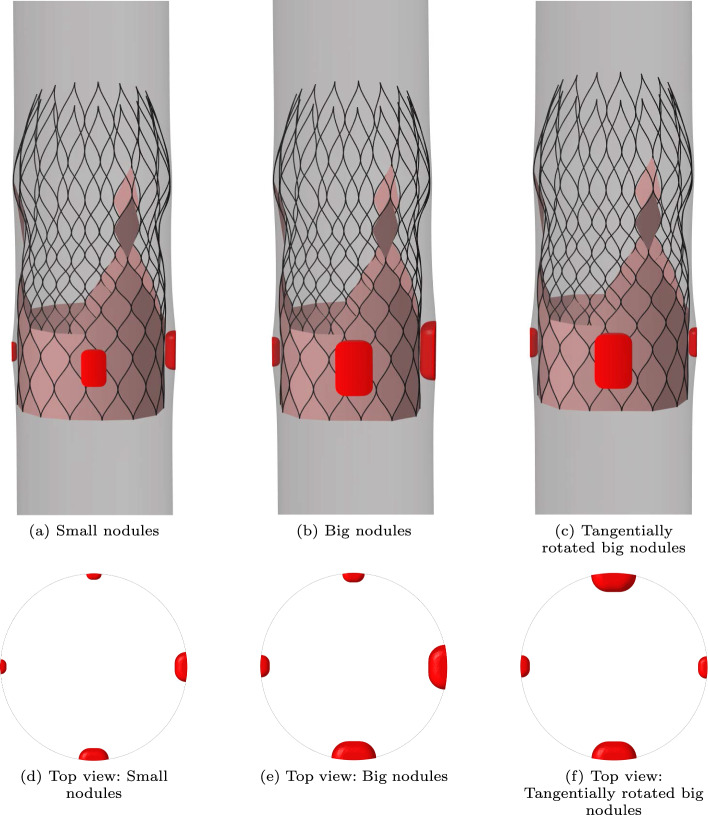
Three test cases of a medium-sized stent (black) deployed inside a hollow cylinder (gray) including multiple calcification nodules (red): **a** small nodules, **b** big nodules, **c** tangentially rotated big nodules. Top view: **d**, **e** **f**

In the experimental results, only one calcification nodule is present in the aortic root geometry. To test whether the ELM predicts the flow rate reasonably well in the case of multiple calcifications, deployment simulations are performed of a medium-sized CoreValve Evolut stent in a hollow cylinder. Inside this cylinder multiple calcification nodules of different sizes are positioned as is shown in Fig. 11, according to the recommendations of (ISO 2021).

**Fig. 12:**
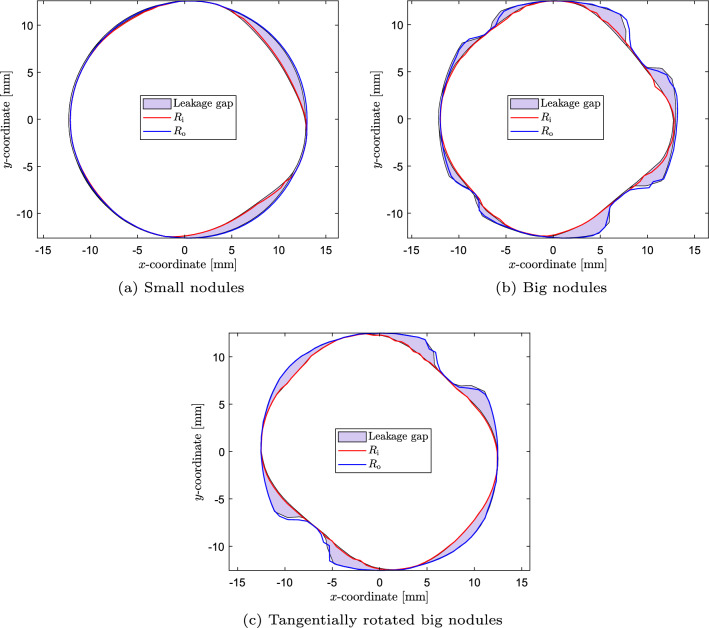
Cross-sectional shape of the gap, used to scale the pressure boundary condition for the ELM (light blue area), and the corresponding estimated ‘annular die’ shape (red and blue lines) for the three test cases: **a** Small nodules, **b** Big nodules, **c** Tangentially rotated big nodules

The output geometry of the deployment simulations is used to construct the leakage volume, which is used as input for both the ELM and the CFD simulations. The regurgitant flow rates are calculated for $\text {MAP}=100$ mmHg. The cross-sectional slices of the gap used to scale the pressure boundary condition for the ELM and the corresponding estimated ‘annular die’ shape for the three different test cases are shown in Fig. 12.

The results are compared to the results of the CFD simulations performed on the same deployed geometries and shown in Table 2.

**Table 2:** Comparison of CFD simulations to the ELM for the cylindrical test case geometries with different nodule sizes and placements

$\text {Test case}$	$\varvec{\textrm{RV}_{\textrm{CFD}}}$ $\text {(ml)}$	$\varvec{\textrm{RV}_{\textrm{ELM}}}$ $\text {(ml)}$
Small nodules	35	32
Big nodules	66	54
Tangentially rotated big nodules	67	73

From this section, it can be concluded that in the case of multiple calcifications, the results between the two models are in good agreement. The maximum error between the two models occurs for the big nodules and equals 18$\%$. However, the ELM results are more sensitive to the placement of the nodules, as shown by the larger difference between the regurgitant volumes for the big nodules and the tangentially rotated big nodules calculated by the ELM compared to the CFD simulations. This might be a result of the larger spatial gradient in gap size for the big nodules due to the different placement of the calcifications, resulting in a larger error in the ELM calculation (see Appendix 3). Additionally, the scaling procedure to find a suitable pressure boundary condition depends on the area and shape of the leakage slice with the smallest maximum gap (see Fig. 6). This leakage slice (and therefore the applied pressure difference) is different for the different test cases using the big nodules (see Fig. 12b and c, where different cross-sectional shapes are obtained since slices at different axial heights are selected in the scaling process), leading to non-identical regurgitant volumes. Therefore, the dependence of the regurgitant volume on the nodule position is a limitation of the ELM.

The ELM computations are much faster than the CFD simulations. To give an indication on the computation time: solving Eq. (1) and calculating the corresponding flow rate takes less than a minute, while the CFD simulations take several hours. This indication excludes the time for the input geometry generation, which is the same for both models.

## Application

In this section the intended application of the ELM model for preoperative risk assessment of paravalvular leakage is highlighted. To this end, deployment simulations are performed of different sizes of a stent based on the CoreValve Evolut in a synthetic average female and a synthetic average male aortic root geometry. A MAP of 100 mmHg is assumed and the ELM is used to calculate the regurgitant flow rates. Furthermore, the simulations of the medium-sized stent in the synthetic average female aortic root geometry are repeated for different degrees of aortic stenosis and the effect of this on the outcomes predicted with the leakage model is investigated.

### Stent sizing

**Fig. 13:**
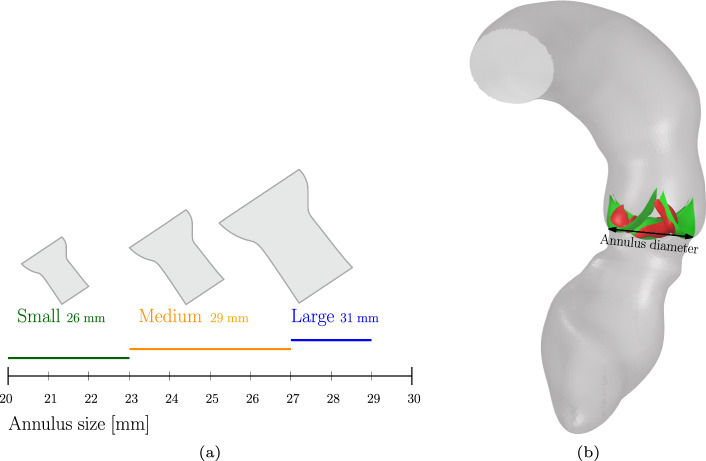
Schematic representation of the different stent sizes used throughout this paper **a**. The diameter of the inflow duct is indicated in the figure, together with the corresponding suitable annulus size ranges for implantation. The annulus size is depicted in **b**

In this paper, three different stent sizes are considered in the deployment simulations, referred to as small, medium and large. These stent shapes are based on the Medtronic CoreValve Evolut stent, where the diameter of the inflow track is different and must be matched with the specific aortic annulus size of the patient. The different stents and their corresponding sizes and corresponding annulus size ranges are schematically represented in Fig. 13a. The annulus diameter is schematically depicted in Fig. 13b.

In this work, the small CoreValve Evolut stent is modeled and scaled in radial direction to obtain the medium and large stent. Therefore, the only parameter varied is the diameter of the inflow track.

**Fig. 14:**
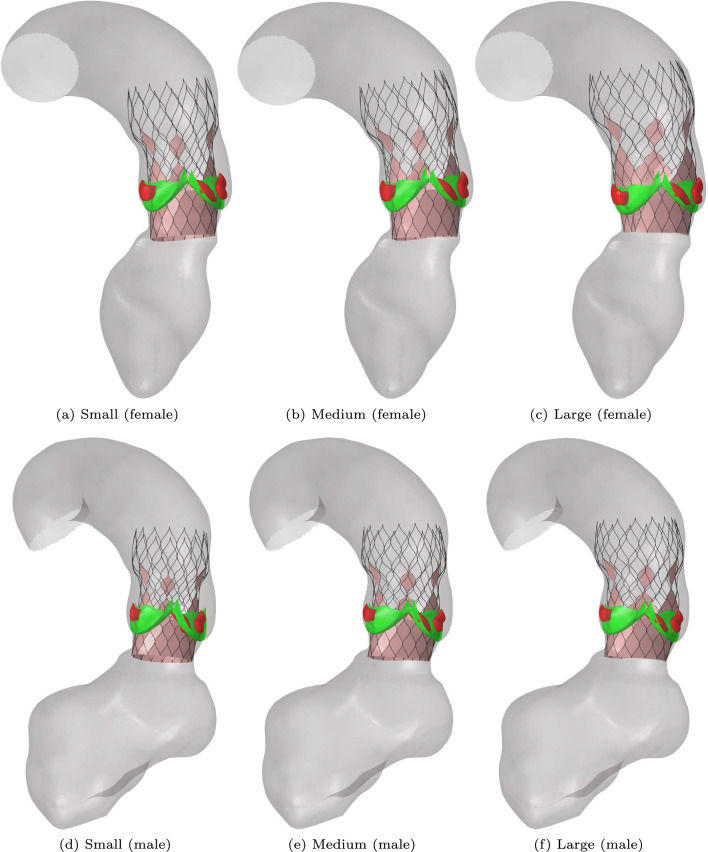
Results of the deployment simulations of different stent sizes inside a synthetic average female aortic root geometry: **a** small, **b** medium, **c** large and a synthetic average male aortic root geometry: **d** small, **e** medium, **f** large

The three different stent sizes depicted in Fig. 13 are deployed in a moderately calcified synthetic average female and male aortic root geometry. The results of the deployment simulations are shown in Fig. 14. The annulus size (as indicated in Fig. 13b) of the average female aortic root is approximately 24 mm, which corresponds to a medium stent (Fig. 13). The annulus size of the average male aortic root is approximately 27 mm, which corresponds to a medium or large stent.

**Fig. 15:**
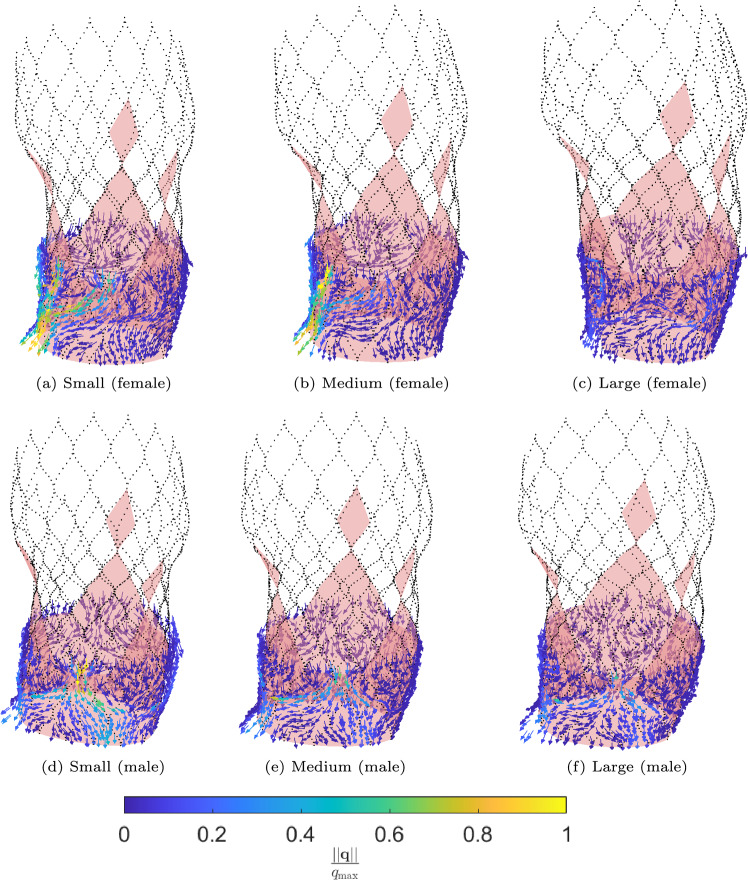
Visualization of the normalized leakage flux in the average synthetic female aortic root geometry obtained from the ELM for different implanted stent sizes: **a** small, **b** medium, **c** large and the average synthetic male aortic root geometry: **d** small, **e** medium, **f** large

The fluid flux $\varvec{q}$ is calculated using the ELM and visualized in Fig. 15. Here, the colors indicate the magnitude of the fluid flux $\varvec{q}$, scaled with $q_{\textrm{max}}=\textrm{max}(||\varvec{q}||)$ of the stent geometry that resulted in the largest regurgitant flow rate. The results are listed in Table 3.

**Table 3:** Regurgitant volumes for different stent sizes implanted in the average female and male synthetic aortic root geometry

$\text {Stent size}$	$\varvec{\textrm{RV}} \text {(ml) average female}$	$\varvec{\textrm{RV}} \text {(ml) average male}$
Small	38	26
Medium	31	16
Large	9	13

The results show that the largest stent leads to the smallest regurgitant volume for both aortic root geometries, as was expected.

According to the PVL classification of Kappetein et al. (2012), the regurgitant volumes for the small and medium stent deployed in the average female aortic root geometry can be classified as moderate leakage, whereas the other regurgitant volumes can be classified as mild leakage. Luraghi et al. (2021) reported that large calcifications may lead to a deformed stent shape or malpositioning of the stented valves after deployment. This means that, even though a larger stent might result in a smaller leakage volume, the final stent shape and stresses induced in the tissue should still be regarded in procedure planning to optimize the postoperative outcome.

### Degree of aortic stenosis

**Fig. 16:**
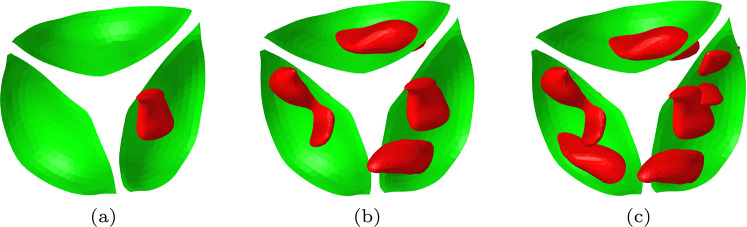
The valves (green) and the included calcification nodules (red) for the three simulated cases of aortic stenosis: **a** Mildly calcified, **b** Moderately calcified and **c** Severely calcified

**Fig. 17:**
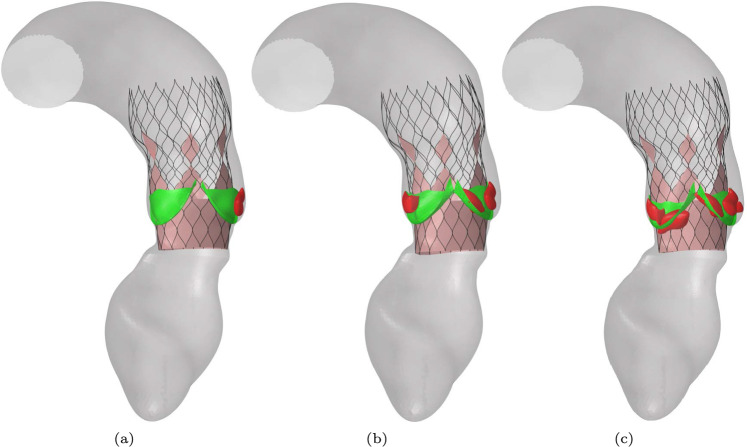
Results of the deployment simulations of the medium stent inside a synthetic average female aortic root geometry: **a** Mildly calcified valves, **b** Moderately calcified valves, c) Severely calcified valves

Deployment simulations are repeated for the medium stent inside the average female aortic root geometry. Now the degree of aortic stenosis is varied by using different numbers of calcification nodules on the valves. Three different degrees of aortic stenosis are simulated, further referred to as mildly, moderately and severely calcified. The valves and the calcifications are shown in Fig. 16. The deployed stents inside the synthetic average female aortic root geometry are shown in Fig. 17.

**Fig. 18:**
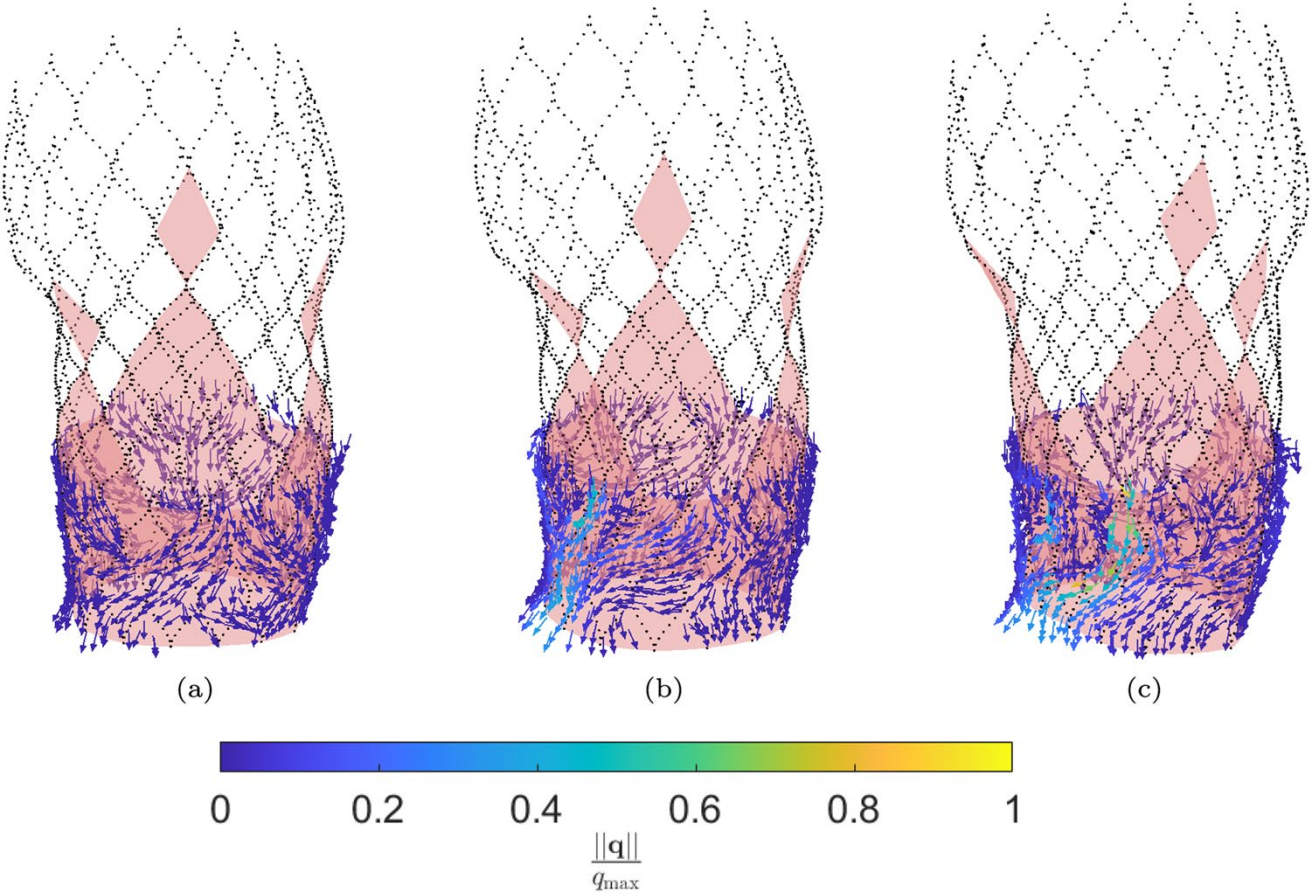
Visualization of the normalized leakage flow in the average synthetic female aortic root geometry obtained from the ELM: **a** Mildly calcified valves, **b** Moderately calcified valves, **c** Severely calcified valves

The fluid flux is calculated using the ELM and visualized in Fig. 18. Here, the colors indicate the magnitude of the fluid flux $\varvec{q}$ scaled with $q_{\textrm{max}}=\textrm{max}(||\varvec{q}||)$ of the severely calcified case. The regurgitant flow rates are again translated into a regurgitant volume, assuming a heart rate of 70 bpm. The results are listed in Table 4.

**Table 4:** Regurgitant volumes for the medium stent implanted in the average female synthetic aortic root geometry for different degrees of stenosis

$\text {Degree of aortic stenosis}$	$\varvec{\textrm{RV}}$ $\text {(ml)}$
Mildly calcified	2
Moderately calcified	31
Severely calcified	48

As expected, the results show that increasing the degree of calcification results in an increased regurgitant volume and thus more severe leakage. This was also found by Luraghi et al. (2021). Using the PVL classification of Kappetein et al. (2012), the mildly and moderately calcified valves result in mild leakage, while the severely calcified vales show moderate leakage.

In conclusion, the results in this section shows the potential of the combination of the deployment model and the ELM for ranking different TAVR stents with respect to the risk of PVL in patient-specific geometries.

## Discussion

The aim of the workflow presented in this paper is to perform fast preoperative risk assessment of PVL, which can be used for procedure planning purposes, such as selecting a suitable stent size for a specific patient. The main advantage of the ELM over existing CFD and FSI simulations is the reduced computation time of the model. In order to make the workflow fast, several assumptions had to be made. The results presented in Sect. 4 showed that the ELM estimates the regurgitant volume well. However, the inherent assumptions reduce the accuracy of the calculated regurgitant volume and the limitations of the ELM will be discussed in this section.

Firstly, the ELM assumes a laminar flow, while in reality, the velocities might become considerably large in the small leakage gaps. To provide a proof of concept of the ELM, results are compared to in vitro experiments and a 3D turbulent CFD model. The experimental model includes one single calcification nodule. To test whether the ELM yields qualitative results when multiple calcifications are present, deployment simulations are performed in an artificial cylindrical aorta including multiple calcification nodules. The ELM results are compared to the 3D turbulent CFD model. The results showed that the ELM is able to give a qualitative prediction of PVL. However, if detailed insight into the hemodynamics of the leakage flow is desired, the ELM should be complemented with CFD simulations. This may be performed consecutively, first a fast selection is performed using the ELM, followed by a detailed analysis of a limited set of cases with CFD. Thus, saving throughput time and minimizing computational cost.

Secondly, in the scaling approach of the pressure boundary condition a circular cross-sectional shape of the stent is assumed in small tangential intervals $\textrm{d}\theta$. This means that errors are introduced in the leakage calculations when the final stent shape deviates from its original circular shape. Since in reality, highly deformed stent shapes induce high stresses in the tissue and improper deployment of the artificial valves, these ‘elliptical’ shapes are undesired. Therefore, these stents should be removed from the treatment options.

Furthermore, the ELM model is based on the thin-film theory. This means that the results are only valid when the spatial gradients in the gap size are small. In reality these spatial gradients can become large for highly calcified valves. The error introduced by large spatial gradients in gap size is studied in Appendix 3. Here, an artificial geometry is designed consisting of ‘pockets’ that introduce a well-defined spatial gradient in gap size. The regurgitant flow rate is calculated using the ELM and a 3D finite element simulation. The results are compared and the error between the ELM and the 3D FEM simulations is plotted for different spatial gradients in gap size. This limitation of the ELM also results in a larger sensitivity to the placement of the calcification nodules, compared to the CFD simulations. Additionally, the scaling procedure to find a suitable pressure boundary condition depends on the area and shape of the leakage slice with the smallest maximum gap. This leakage slice (and therefore the applied pressure difference) thus differs for different calcification positions, leading to non-identical regurgitant volumes.

It is important to note that, both, the CFD simulations and the ELM are highly sensitive to the gap size in the leakage volume, as the flow rate is proportional to $R_{\textrm{o}}^{4}$ and $R_{\textrm{i}}^{4}$. Consequently, the accuracy of the calculated regurgitant volume significantly depends on the quality of the segmented aortic root geometry and the mesh. While the ELM results align well with the experimental data and CFD simulations, further validation on a large cohort of patients is necessary. This validation should encompass diverse patient-specific conditions, including variations in the geometrical aspects of the aortic root, to confirm the accuracy of the model’s predictions. Additionally, a transient 3D FSI model should be developed to model the hemodynamic flow during the full cardiac cycle of the heart including the effect of the contraction of the ventricle and the coronary arteries.

**Fig. 19:**
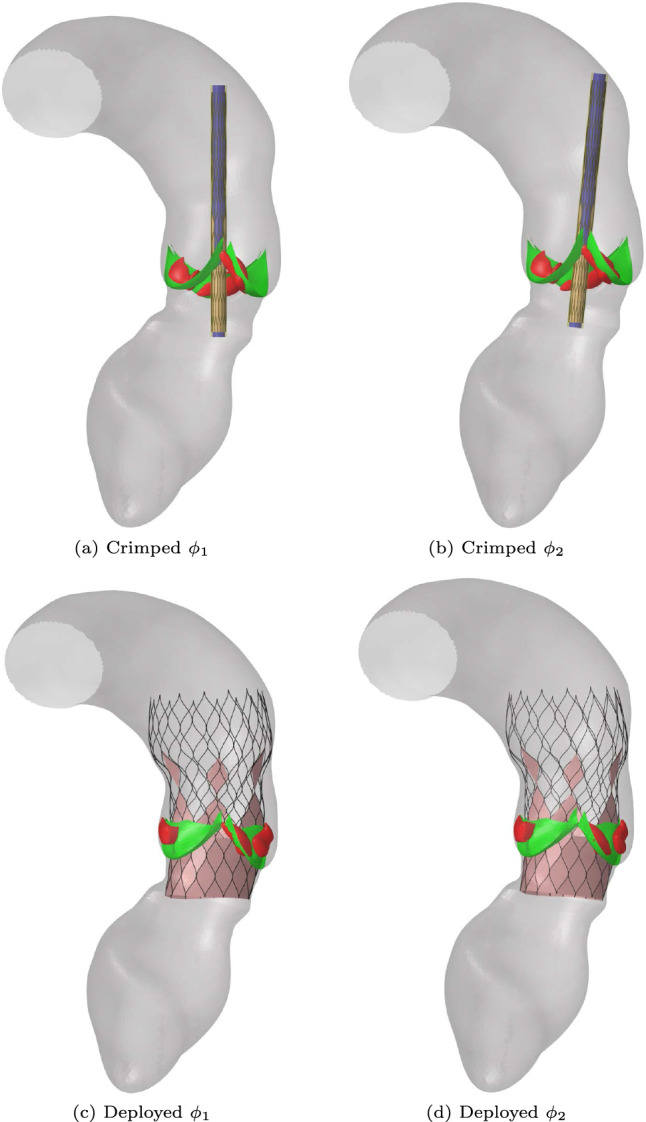
Results of the deployment simulations of the medium stent inside a synthetic average female aortic root geometry using two different implantation angles and the final deployed stent inside the aortic root geometry

Finally, results showed that the calculated regurgitant volume is highly dependent on the patient-specific features of the aortic root geometry, the position and shape of the calcification nodules and the deployment strategy, as was previously reported by Morganti et al. (2016). To give an example of the influence of the deployment strategy we performed two simulations of the medium stent in the moderately calcified female aortic root geometry. The only difference between the two simulations is the implantation angle $\phi$ as is shown in Fig. 19.

The corresponding regurgitant volumes, calculated using the ELM, are 41 ml for $\phi _{1}$ and 31 ml for $\phi _{2}$. This result shows that the deployment strategy may have a non-negligible effect on the postoperative outcome and the presented workflow can be used to optimize the deployment strategy for individual patients. However, for both, the deployment model and the ELM validation on a large cohort of real patients is necessary and a topic for future work.

## Conclusions

In this paper, a numerical framework is presented to perform fast preoperative risk assessment of paravalvular leakage, using a novel simplified leakage model. This model can be used to calculate the regurgitant volume after TAVR at reduced cost.

To generate a patient-specific input for the leakage model, an explicit finite element model is used to simulate the deployment of a self-expandable stent, based on the CoreValve Evolut, inside an aortic root geometry. Using the result from the 3D deployment model, leakage slices at different axial heights in the region of interest (close to the valves) are obtained to reconstruct the leakage volume. These leakage slices are also used to scale the MAP to obtain a pressure boundary condition that is consistent with the ‘flow through an orifice’ problem characteristic for PVL.

A proof-of-concept analysis of the efficient leakage model (ELM) is performed by comparing the results to in vitro experiments. To test whether the ELM gives reliable results in scenarios where multiple calcifications are present, the regurgitant volume is calculated for a medium CoreValve Evolut stent deployed in a cylindrical test aortic root geometry including multiple calcification nodules of different sizes. The results of the ELM model are compared to 3D CFD simulations. The comparison revealed reasonably close regurgitant volume values, providing confidence in the ELM’s ability to offer a qualitative indication of the risk of paravalvular leakage.

Finally, preoperative risk assessment results are presented to show the intended clinical application of the presented workflow. To this end, the regurgitant volume is calculated for stents of different sizes deployed in a synthetic average male and female aortic root geometry. This is repeated for the medium-sized stent in the synthetic female aortic root geometry with different degrees of calcifications. The results show the potential of the presented workflow to be used to obtain valuable preoperative insights in the risk of paravalvular leakage. The presented simplified leakage model is less computationally expensive compared to CFD simulations. Furthermore, the ELM is less susceptible to convergence problems in the narrow leakage gaps. If the ELM gives an indication of high risk of paravalvular leakage under certain patient-specific circumstances, CFD simulations could be used to give additional insights into the hemodynamics of the problem if this is desired.

This paper showed that the ELM can be used to give a fast indication of the risk of PVL with sufficient accuracy. In addition to the calculation of the regurgitant volume, the leakage path along which blood can flow past the stent, can be visualized. Due to the reduced computation time of the ELM compared to currently existing CFD and FSI model, the presented workflow can be a valuable tool for clinicians to use in procedure planning. In this regard, the ELM can be used to rank different TAVR designs and sizes and optimize the deployment strategy with respect to the risk of PVL for patient-specific aortic root geometries. The results presented in this paper are limited to the CoreValve Evolut stent. However, the model is general enough that other stent designs can easily be included. However, for both, the deployment model and the ELM validation on a large cohort of real patients are necessary and an important topic for future work.

## Figures and Tables

**Fig. 20:**
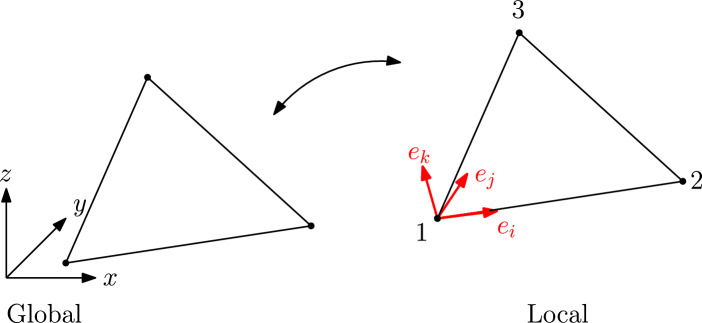
Schematic representation of a shell element with the nodes numbered as indicated by the black dots and the local coordinate system in red

**Fig. 21:**
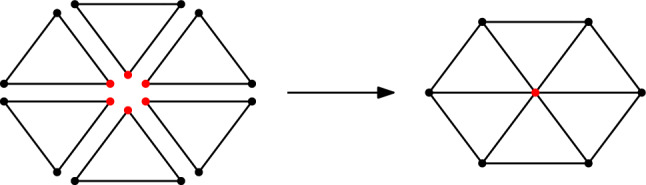
Schematic representation of six connecting elements sharing one common node (red). Every shell element has its own local coordinate system and therefore there are six contributions in the common node to the vector of the fluid flux $\varvec{q}$

**Fig. 22:**
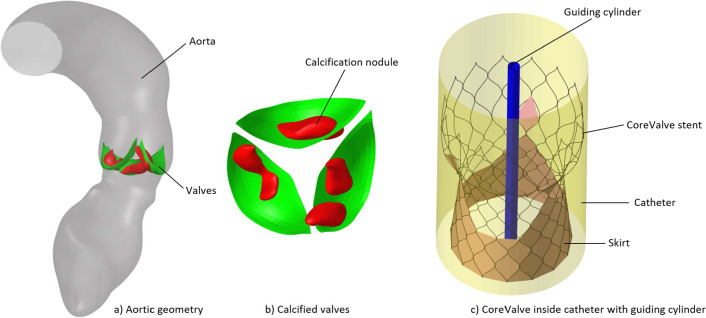
Schematic representation of the different geometries used to model the deployment of a self-expandable stent

**Fig. 23:**
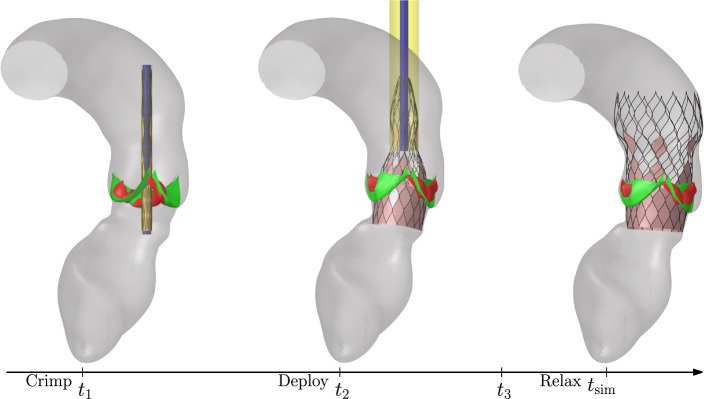
Schematic workflow representation of the deployment simulations in time. From left to right: stent crimped inside the catheter ($t\le t_{1}$), stent is released from catheter ($t_{1} < t \le t_{3}$), final shape of deployed stent inside the aorta ($t_{\textrm{sim}}$)

**Fig. 24:**
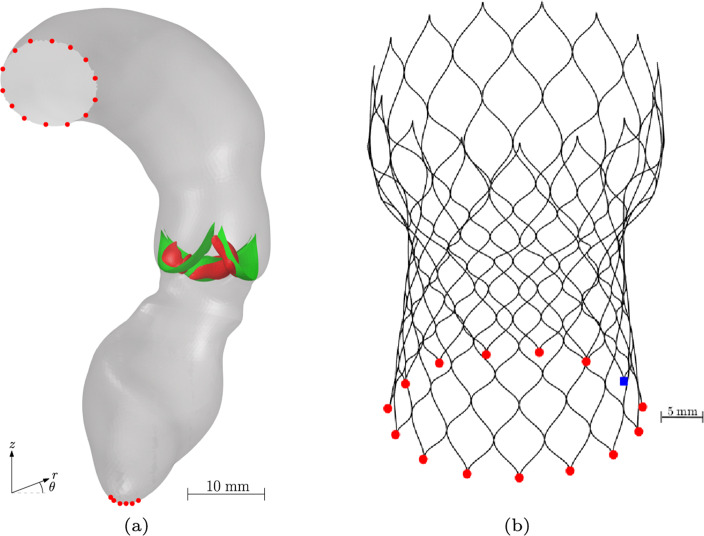
**a** Nodes on the aorta to which a Dirichlet boundary condition is applied to prescribe the displacement. **b** Stent nodes to which a Dirichlet boundary condition is applied to prescribe the displacement

**Fig. 25:**
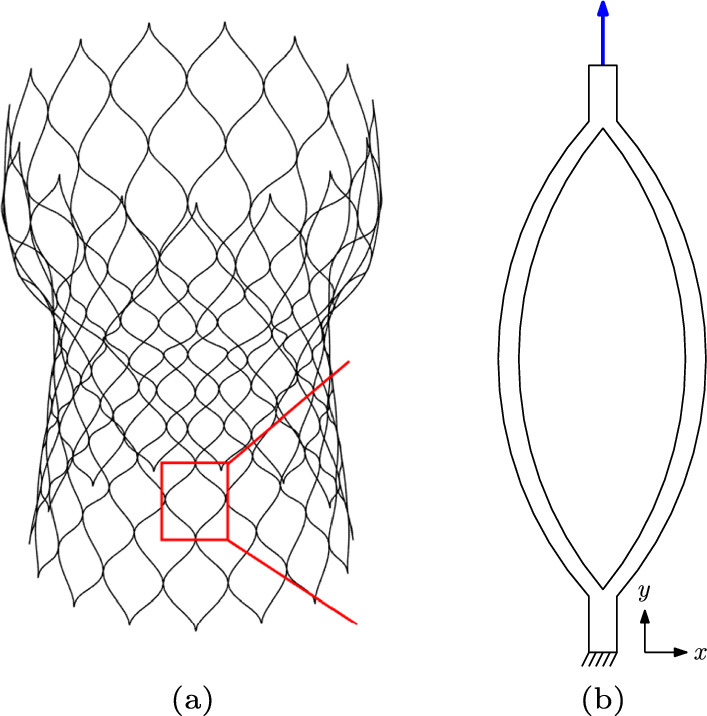
Schematic representation of a single cell of the stent displaced vertically

**Fig. 26:**
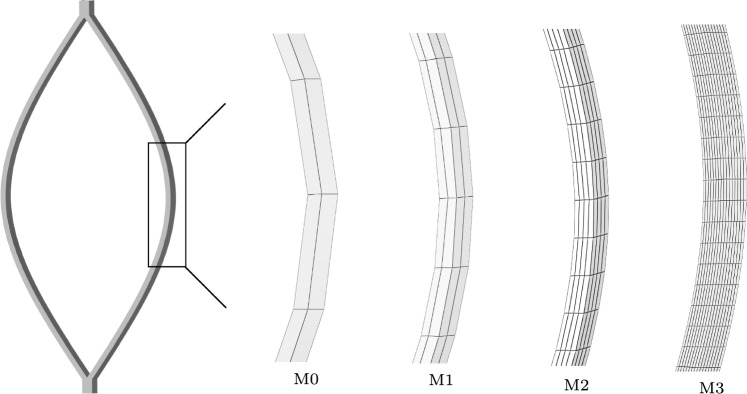
Single cell of the stent with magnified figures of the different 3D meshes used in the convergence study

**Fig. 27:**
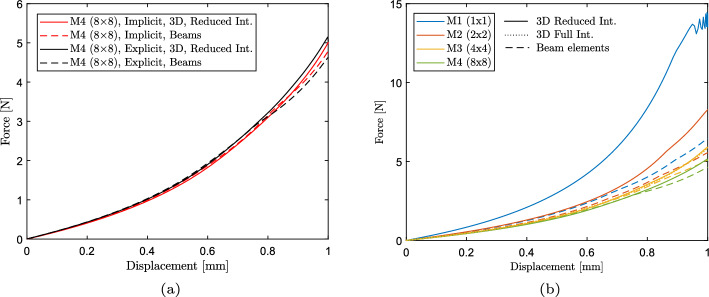
Results of convergence tests of the single cell under tension. Results of implicit and explicit simulations are compared **a** as well as explicit simulation results using 3D elements and beam elements **b**

**Fig. 28:**
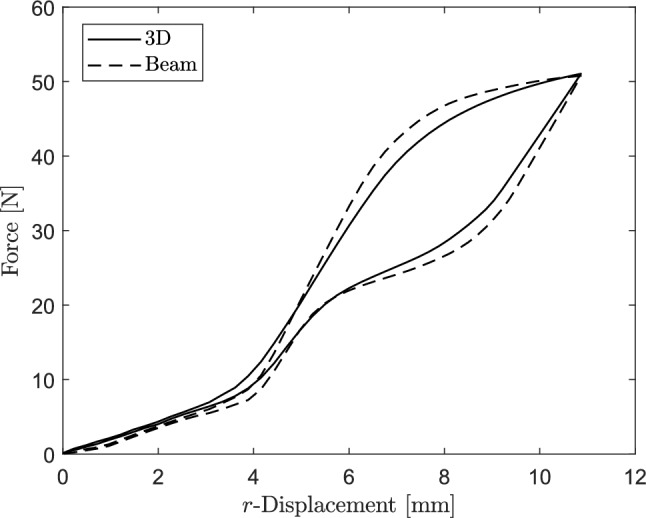
Force displacement curves for radial crimping tests on the CoreValve Evolut stent modeled with beam elements and 3D elements

**Fig. 29:**
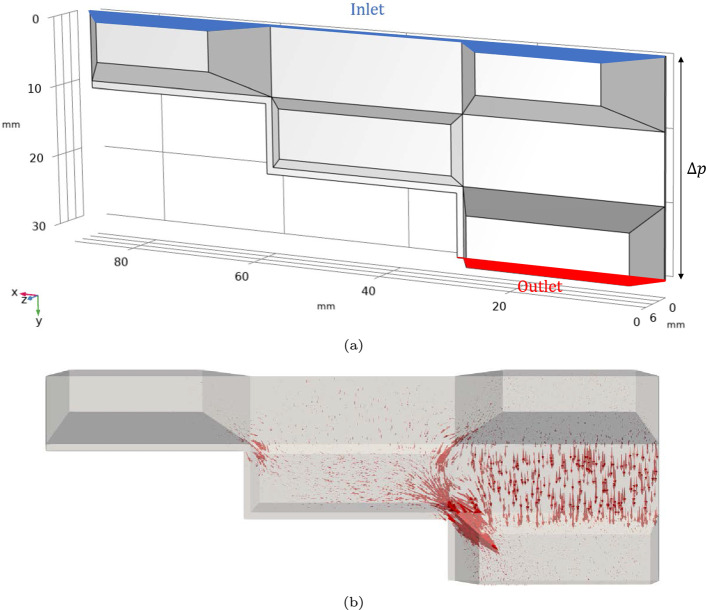
**a** Test geometry used to get an indication of the error made by the ELM due to large spatial variations in gap size. The inlet is the top surface (blue) of the geometry, whereas the outlet is the bottom surface of the geometry (red). **b** Front view of the test geometry with arrows indicating the flow obtained from the 3D FEM simulations

**Fig. 30:**
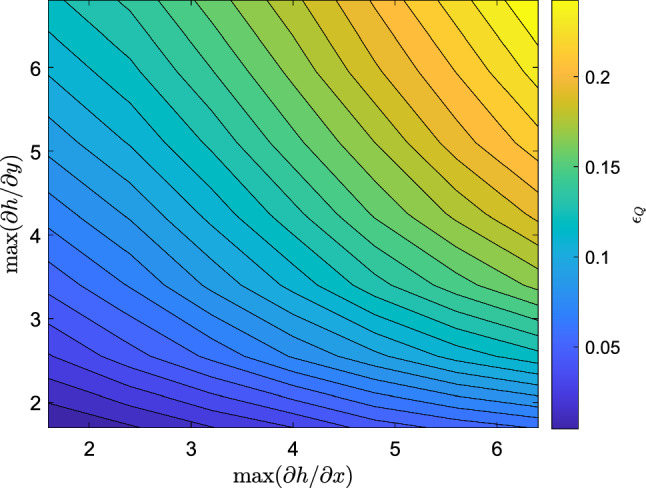
The error between the flow rate calculated by the ELM and the flow rate calculated by the 3D FEM model, for different gradients of the gap size *h* in *x*- and *y*-direction. This figure contains the results of 49 simulations

**Fig. 31:**
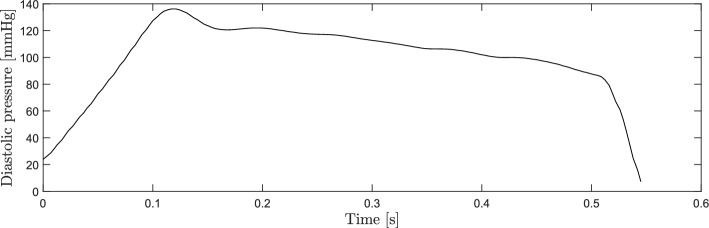
Applied transient pressure boundary condition $p_{\textrm{in}}(t)$

**Fig. 32:**
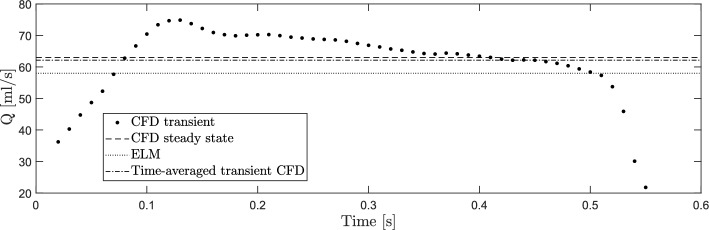
Comparison of the calculated regurgitant flow rate for the ‘small nodules’ artificial case obtained with the transient and steady-state CFD simulations and the result of the ELM model. The dash-dot line represents the time-averaged flow rate of the transient CFD simulation

**Fig. 33:**
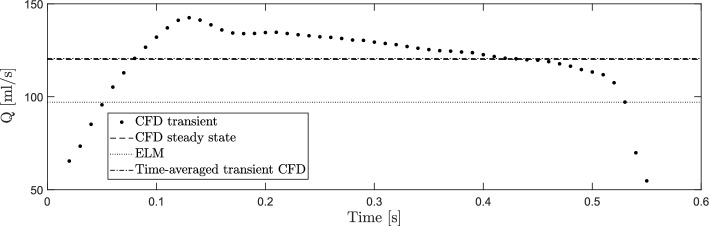
Comparison of the calculated regurgitant flow rate for the ‘big nodules’ artificial case obtained with the transient and steady-state CFD simulations and the result of the ELM model. The dash-dot line represents the time-averaged flow rate of the transient CFD simulation

## Appendix 1: Finite element implementation of the efficient leakage model

In this appendix, details on the finite element implementation of the efficient leakage model can be found.

### Flow rate calculation

The weak formulation of the Reynolds equation for pressure driven, incompressible, viscous Poiseuille flow (Eq. (1)) can be derived by multiplying the equation with a test function and integrating over the domain using partial integration and using Gauss’ theorem (Hughes 2000). The weak form of this problem can now be formulated as follows: find *p* such that:

5 $$\begin{aligned} \int _{\Omega }\varvec{\nabla }v\cdot \Big (\frac{h^{3}}{12\mu }\varvec{\nabla }p\Big )\;\textrm{d}\Omega - \int _{\Gamma } v\frac{h^{3}}{12\mu }\varvec{n}\cdot (\varvec{\nabla }p)\;\textrm{d}\Gamma = 0, \end{aligned}$$

for all admissable test functions *v*. Here, $\Omega$ indicates the fluid domain and $\Gamma$ the domain boundaries.

After partitioning of the system matrix, the following system of equations is solved to obtain the unknown pressures *p*:

6 $$\begin{aligned} \begin{bmatrix} \varvec{K}_{\textrm{uu}} &{} \varvec{K}_{\textrm{up}} \\ \varvec{K}_{\textrm{pu}} &{} \varvec{K}_{\textrm{pp}} \end{bmatrix} \begin{bmatrix} \varvec{p}_{\textrm{u}} \\ \varvec{p}_{\textrm{p}} \end{bmatrix} = \begin{bmatrix} \varvec{f}_{\textrm{u}} \\ \varvec{f}_{\textrm{p}} \end{bmatrix}, \end{aligned}$$

where the subscript u stands for unknown, and p for prescribed. The u-part can be solved to obtain the unknown pressures:

7 $$\begin{aligned} \varvec{K}_{\textrm{uu}} \varvec{p}_{\textrm{u}} = \varvec{f}_{\textrm{u}} - \varvec{K}_{\textrm{up}}\varvec{p}_{\textrm{p}}. \end{aligned}$$

On the inflow and outflow boundary of the shell mesh, the pressures $\varvec{p}_{\textrm{p}}$ are prescribed using a Dirichlet boundary condition. Furthermore, the prescribed right hand side in all nodes $\varvec{f}_{\textrm{p}}$, corresponds to the second term in Eq. (5). For the inflow and outflow boundary of the domain, this term equals the flow rate for a viscous Poiseuille flow. This means the flow rate can be obtained by solving the following equation:

8 $$\begin{aligned} \varvec{f}_{\textrm{p}} = \varvec{K}_{\textrm{pu}}\varvec{p}_{\textrm{u}} + \varvec{K}_{\textrm{pp}}\varvec{p}_{\textrm{p}}. \end{aligned}$$

The flow rate can now be calculated by summation of all the contributions of $\varvec{f}_{\textrm{p}}$ on the Dirichlet boundary:

9 $$\begin{aligned} Q = \sum _{i=1}^{N_{\textrm{nodes}}} \varvec{f}_{\textrm{p}}(\varvec{n}_{\textrm{in}}(i)), \end{aligned}$$

where $N_{\textrm{nodes}}$ equals the number of nodes on the inflow boundary of the domain and $\varvec{n}_{\textrm{in}}$ is a vector containing the node numbers of the nodes on the top boundary of the shell mesh. Note that replacing $N_{\textrm{nodes}}$ by the number of nodes on the outflow boundary of the domain and $\varvec{n}_{\textrm{in}}$ by $\varvec{n}_{\textrm{out}}$ should yield the same flow rate.

### Shell element implementation

The Reynolds equation for pressure driven, stationary, incompressible, viscous Poiseuille flow is solved in the simplified leakage model:

10 $$\begin{aligned} \varvec{\nabla }\cdot \Big [-\frac{h^{3}}{12\mu }\varvec{\nabla }p\Big ] = 0. \end{aligned}$$

The weak form of Eq. (10), given in Eq. (5), is solved on element level in a local coordinate system, defined in the plane of the shell elements such that the thickness of the gap is always normal to this plane. This is schematically represented in Fig. 20a. The coordinates of the nodes of the shell element in the global coordinate system are represented by the vector $\varvec{x}_{0}$. The unit vectors of the local coordinate system $\varvec{e}$ can now be defined as follows:

11 $$\begin{aligned}&\varvec{e}_{i} = \frac{\varvec{x}_{0}(2)-\varvec{x}_{0}(1)}{\Vert \varvec{x}_{0}(2)-\varvec{x}_{0}(1) \Vert }, \end{aligned}$$

12 $$\begin{aligned}&\varvec{e}_{j} = \frac{\big (\varvec{x}_{0}(2)-\varvec{x}_{0}(1)\big ) \times \big (\varvec{x}_{0}(3)-\varvec{x}_{0}(1)\big )}{\Vert \big (\varvec{x}_{0}(2)-\varvec{x}_{0}(1)\big ) \times \big (\varvec{x}_{0}(3)-\varvec{x}_{0}(1)\big ) \Vert }, \end{aligned}$$

13 $$\begin{aligned}&\varvec{e}_{k} = \varvec{e}_{i} \times \varvec{e}_{j}, \end{aligned}$$

where 1, 2, 3 indicate the components of the coordinate vector.

The coordinates, expressed in the local coordinate system of the shell elements, $\varvec{x}_{0e}$, can be obtained by performing the following transformation:

14 $$\begin{aligned}&\varvec{x}_{0e}(1) = \varvec{x}_{0}\cdot \varvec{e}_{i}, \end{aligned}$$

15 $$\begin{aligned}&\varvec{x}_{0e}(2) = \varvec{x}_{0}\cdot \varvec{e}_{j}, \end{aligned}$$

16 $$\begin{aligned}&\varvec{x}_{0e}(3) = \varvec{x}_{0}\cdot \varvec{e}_{k}. \end{aligned}$$

After transformation to the local coordinate system, the element contributions to the system matrix can be calculated following the standard Finite Element Method approach (Hughes 2000). These are used to construct the global system matrix.

For visualization purposes of the blood flow, the fluid flux $\varvec{q}=-h^{3}(\varvec{\nabla }p)/12\mu$ is calculated, using the pressure obtained from solving the weak form of Eq. (1). In the nodes that are shared by multiple elements, there will be contributions to the fluid flux $\varvec{q}$ from multiple local coordinate systems belonging to the different elements, as is schematically depicted in Fig. 21.

Since $\varvec{q}$ is a vector, the results obtained in the local coordinate system in the nodes of the shell element $\varvec{q}_{e}$ need to be transformed back to the global coordinate system to obtain the 3D global vector $\varvec{q}$. This is done by performing the following transformation:

17 $$\begin{aligned} \varvec{q} = \varvec{T}\varvec{q}_{e}, \end{aligned}$$

where $\varvec{T}$ is the transformation matrix:

18 $$\begin{aligned} \varvec{T} = \begin{bmatrix} \varvec{e}_{i}(1) &{} \varvec{e}_{k}(1)\\ \varvec{e}_{i}(2) &{} \varvec{e}_{k}(2)\\ \varvec{e}_{i}(3) &{} \varvec{e}_{k}(3) \end{bmatrix}. \end{aligned}$$

The contributions of $\varvec{q}$ from the different elements in the shared node are averaged in the global coordinate system.

## Appendix 2: Deployment simulations

Deployment simulations are performed to generate an input geometry for the ELM. In this appendix, more details on the numerical model can be found.

### Problem description

The stent, and the skirt surrounding the stent, are crimped into the catheter. Inside the catheter, a guiding cylinder is added to keep the outflow part of the stent device from coming into contact with itself. After positioning inside the aortic annulus, the catheter covering the crimped device is removed to deploy the stent. A schematic representation of the different parts of the model is shown in Fig. 22. Here, the aortic valve leaflets are indicated in green and the calcifications in red.

### Material properties

Auricchio’s constitutive model for Nitinol is used to model the stent behavior (Auricchio and Taylor 1997). The material parameters are adopted from Morganti et al. (2016) and listed in Table 5. Simplified linear elastic material models are used to model the catheter and the guiding cylinder, whereas a hyperelastic Neo-Hookean material model is used to model the tissue. The material properties used in these models are taken from literature (Morganti et al. 2014; Morganti et al. 2016) and are listed in Table 6. For the Neo-Hookean material model $C_{10}=G/2$ with $G=E/\big (2(1+\nu )\big )$ with *G* the shear modulus, *E* the Young’s modulus and $\nu$ the Poisson’s ratio. Due to the large mismatch in stiffness values between the tissue and the calcifications, the calcifications are modeled as rigids. The thickness of the shell elements of the aortic wall and the valves are set to 2.5 and 0.5 mm, respectively (Morganti et al. 2014). The material properties for the catheter and guiding cylinder are dummy parameters mimicking a rigid material.

**Table 5:** Nitinol material properties used for the constitutive model for the stent adopted from Morganti et al. (2016)

Parameter	Description	Value
$E^{\textrm{A}}$	Elastic modulus austenite	51700 MPa
$E^{\textrm{M}}$	Elastic modulus martensite	47800 MPa
$\nu$	Poisson’s ratio	0.3
$\sigma _{\textrm{L}}^{\textrm{S}}$	Start of transformation loading	600 MPa
$\sigma _{\textrm{L}}^{\textrm{E}}$	End of transformation loading	670 MPa
$\sigma _{\textrm{U}}^{\textrm{S}}$	Start of transformation unloading	288 MPa
$\sigma _{\textrm{U}}^{\textrm{E}}$	End of transformation unloading	254 MPa
$\epsilon ^{\textrm{L}}$	Maximum transformation strain	6.3$\%$
$\alpha$	Material parameter measuring the difference in response between tension and compression	0.2

**Table 6:** Material properties used for the different components; $\rho$ is the density, *E* is Young’s modulus, and $\nu$ is Poisson’s ratio

Component	$\rho (\text {kg}/\text {mm}^{3})$ ()	*E* (MPa)	$\nu$ (-)
Aorta, leaflets	1e–9	2	0.45
Skirt	1e–9	2	0.45
Catheter, guiding cylinder	1e–9	50000	0.3

### Workflow and boundary conditions

The workflow of the numerical model is schematically depicted in Fig. 23. First the stent is crimped into the catheter (yellow). A guiding cylinder is added (blue) to prevent the outflow part of the stent from coming into contact with itself. Contact is defined between the stent and the catheter and the stent and the guiding cylinder. As a next step, the catheter and guiding cylinder are removed and the stent is deployed into the synthetic patient anatomy. Additional contact is now defined between the relevant component pairs. This means that contact is defined between the following component pairs: stent-valves, stent-calcifications, stent-aorta, aorta-valves, aorta-calcifications.

The simulation is separated in different phases: the crimping phase $0< t \le t_{1}$, deployment phase with fixed crimped radius of the catheter $t_{1} < t \le t_{2}$, deployment phase during which the catheter radius is slightly increased $t_{2} < t \le t_{3}$, relaxation phase during which the stent is fully deployed inside the aorta $t > t_{3}$. The node sets to which the boundary conditions are applied are schematically depicted in Fig. 24.

*Stent*: During the crimping step, the nodes on the inflow boundary of the stent (see Fig. 24b) are restricted to move in the tangential and axial direction. To enable the stent to deform in all directions, only one node of the inflow boundary of the stent (indicated by the blue square presented in Fig. 24b) is restricted to move in the tangential and axial direction during the deployment step. This is sufficient to avoid rigid body motions, since the stent makes contact with the catheter, the leaflets and the aorta wall upon deployment.

*Guiding cylinder*: All nodes of the guiding cylinder are fixed in place during the crimping step and moved in *z*-direction in the deployment step.

*Catheter*: A displacement function is prescribed to all nodes of the catheter during the crimping and deployment step to assure gradual unfolding. Furthermore, the catheter is moved in *z*-direction during the deployment step.

The applied Dirichlet boundary conditions are summarized in Table 7. Here, *R* is the magnitude of the radial crimping in [mm] depending on the diameter of the stent and the final (crimped) diameter of the stent and *Z* is the magnitude of the vertical displacement in [mm]. In the simulations presented in this paper, the stent is crimped to a diameter of 4 mm. The total pseudo-time $t_{\textrm{sim}}$ of the deployment simulations is 0.04 s. For this pseudo-time the simulations are sufficiently fast, but no vibrations are introduced to the stent after deployment.

**Table 7:** Dirichlet boundary conditions applied to the different components in the deployment simulations to prescribe displacements

Component	Prescribed displacement	Function
Aorta (red nodes Fig. 24a)	$u_{r}$, $u_{\theta }$, $u_{z}$	$0\; \forall \; t$
Stent (red + blue (square) nodes Fig. 24b)	$u_{\theta }$, $u_{z}$	$0 \; \text {for} \; 0<t\le t_{1}$
Stent (blue (square) node Fig. 24b)	$u_{\theta }$, $u_{z}$	$0 \; \text {for} \; t > t_{1}$
Guiding cylinder (all nodes)	$u_{r}$, $u_{\theta }$	$0 \; \forall \; t$
	$u_{z}$	$0 \; \text {for} \; 0<t\le t_{1}$
		$$\frac{2Zt}{t_{\textrm{sim}}} \; \text {for} \; t_{1}<t\le t_{3}$$
		$$0 \; \text {for} \; t_{3}<t\le t_{\textrm{sim}}$$
Catheter (all nodes)	$u_{\theta }$	$0\; \forall \; t$
	$u_{r}$	$-\frac{4Rt}{t_{\textrm{sim}}} \; \text {for} \; 0<t\le t_{1}$
		$$0 \; \text {for} \; t_{1}<t\le t_{2}$$
		$$\frac{8Rt}{t_{\textrm{sim}}} \; \text {for} \; t_{2}<t\le t_{3}$$
		$$0 \; \text {for} \; t_{3}<t\le t_{\textrm{sim}}$$
	$u_{z}$	$0 \; \text {for} \; 0<t\le t_{1}$
		$$\frac{2Zt}{t_{\textrm{sim}}} \; \text {for} \; t_{1}<t\le t_{3}$$
		$$0 \; \text {for} \; t_{3}<t\le t_{\textrm{sim}}$$

### Verification

To verify the numerical implementation of the deployment model, this section shows the results of convergence tests performed on a single cell of the stent. Furthermore, a radial crimping test is performed on the CoreValve Evolut stent modeled with 3D and beam elements to show the validity of the beam approach.

#### Periodic stent part simulations

A vertical displacement is prescribed to a periodic element of the stent at the top of the strut connection, as is schematically shown in Fig. 25. The force displacement curves for the periodic element, or single cell, meshed with reduced integration 3D linear hexahedral elements and 1D beam elements are compared.

The reference mesh $\textrm{M}3$ consists of 80 elements over the curved length of the periodic cell and 8 elements over the width and thickness of the cell. To study mesh convergence, the mesh is uniformly refined in all directions. The 3D meshes used in this study are shown in Fig. 26 and reported in Table 8.

**Table 8:** Meshes used in the convergence study on the single cell

$\text {Mesh}$	# beam elements	# 3D elements
M1	23 (10)	26 (10, 1 × 1)
M2	44 (20)	192 (20, 2 × 2)
M3	88 (40)	1536 (40, 4 × 4)
M4	176 (80)	12288 (80, 8 × 8)

() indicate the number of elements over the curved edge and the number of elements over the cross-section in the 3D models

The 3D results are compared to the results using beam elements. First, implicit versus explicit results are compared for mesh $\textrm{M}4$ in Fig. 27a. The implicit simulations are performed in Abaqus.^8^ For the implicit simulations it is verified that the load step size is small enough to obtain converged results. The load is applied in 10 load steps.

This figure shows that the results for the implicit and explicit simulations are approximately equal. The difference in calculated force at the final displacement for the beam elements and the 3D elements is 3.6 and 2.7$\%$, respectively. Since an explicit formulation is more robust in nonlinear contact problems, the explicit method is used for the simulations and results for 3D elements are compared to the results using beam elements for the different meshes. Figure 27b shows that the results for 3D elements and beam elements converge upon mesh refinement. The error in calculated force between the 3D elements and beam elements of mesh $\textrm{M}4$ at the final displacements is 10$\%$. Due to the inability of the beam elements to capture the strut connections properly, the results of the beam elements will always be an approximation of the 3D results. However, in this paper the deployment simulations are used to perform a preoperative risk assessment for PVL and for this purpose the error of 10$\%$ is assumed to be small enough. For completeness, a 3D simulation is also performed with a full integration element and mesh $\textrm{M}3$. The results using reduced or full integration are in good agreement. Since the results for 3D elements and beam elements give approximately the same result upon mesh refinement, but beam elements are significantly less computationally demanding, beam elements are used in the remainder of this paper to model the stent.

### Radial crimping test

The deployment model is used to perform a radial crimping test to show the validity of the beam element approach. To this end, the CoreValve Evolut stent is crimped inside the catheter and released again. The stent is modeled using 3D elements and beam elements, using a mesh with 21 elements over the curved edge of each ‘loop’ and 4x4 elements over the cross-section of the struts in the 3D model. The force displacement curves of both element types are compared and the results are shown in Fig. 28.

Figure 28 shows a relatively good agreement between the force displacement curves for the different element types. Since beam elements significantly reduce the computational costs, they are used to model the stent in the deployment model to generate the geometrical input for the ELM.

## Appendix 3: Error estimation for increasing spatial gradients in gap size

Since the ELM is based on the thin-film approximation, the method is only valid under the assumption of small spatial variations in gap size. To get an indication of the error made by the ELM due to the variations in gap size, a test geometry is presented consisting of ‘pockets’ with a well-defined gap size. The geometry is designed such that the spatial gradient in gap size can be easily varied and is well defined. The flow rate through the test geometry is calculated using 3D FEM simulations performed in COMSOL^9^ and compared to results from the ELM model.

### Problem description

The initial geometry used to test the influence of increasing spatial gradients in gap size on the accuracy of the ELM model is shown in Fig. 29(a). The geometry consists of multiple ‘pockets’ of a certain thickness to introduce a spatial gradient in gap size in *x*-direction. Over the axial length of the domain, pockets with different thicknesses are positioned to also introduce a spatial gradient of the gap size in *y*-direction. The gradient of the gap size in *x*- and *y*-direction is increased, by decreasing the *x* and *y* length of the geometry domain. This is done to create 49 geometries with different maximum values $\textrm{max}(\partial h/\partial x)$ and $\textrm{max}(\partial h/\partial y)$. The dimensions of the initial geometry are roughly based on the dimensions of the leakage volume obtained from the deployment simulations of a medium stent inside the severely calcified female synthetic aortic root geometry, discussed in Sect. 5. By doing this, the gap sizes of the test geometry are representative for PVL problems.

A pressure difference is applied over the top and the bottom of the domain, to introduce a pressure driven flow from the top surface (inlet) to the bottom surface (outlet).

### Numerical method

FEM simulations are performed on the 3D geometry, where the mass and momentum balance are solved for an incompressible fluid under the assumption that inertia can be neglected:

$$\begin{aligned}&\varvec{\nabla }\cdot \varvec{v} = 0, \quad{} & {} \text {in}\;\Omega \\ -&\varvec{\nabla }\cdot \varvec{\sigma }=\varvec{0}, \quad{} & {} \text {in}\;\Omega \\ \end{aligned}$$

where, $\varvec{v}$ is the fluid velocity and $\varvec{\sigma }=-p\varvec{I} + 2\mu \varvec{D}$ is the Cauchy stress tensor, with $\varvec{I}$ the unit tensor and $\varvec{D} = (\varvec{\nabla }\varvec{v} + \varvec{\nabla }\varvec{v}^{T})/2$ the rate of deformation tensor. The traction $\varvec{t}=\varvec{\sigma }\cdot \varvec{n}$, with $\varvec{n}$ the unit surface normal, on the top surface of the domain is prescribed as 13.32 kPa, whereas the traction on the bottom surface of the domain is zero. This boundary condition generates a pressure difference over the inlet (indicated in blue) and outlet (indicated in red) as indicated in Fig. 29a. A no-slip condition, $\varvec{v}=\varvec{0}$, is applied on all other boundaries. For the velocity and the pressure isoparametric, tetrahedral $P_{2}P_{1}$ (Taylor-Hood) elements are used. The flow in the test geometry can now be visualized as is shown in Fig. 29b. The flow rate is obtained by integration of the velocity over the outlet surface. Additionally, the ELM model is used to calculate the flow rate, following the approach of Sect. 2. To this end, a shell mesh is constructed, using linear elements, in the middle of the gap of the 3D geometry.

### Results

In total 49 simulations for 49 geometries with different maximum spatial gradients of the gap size *h* in *x*- and *y*-direction are performed. Results of the flow rate of the 3D FEM simulations are compared to the results of the ELM. The error between the outcome of the 3D FEM simulations and the ELM is calculated as follows:

19 $$\begin{aligned} \epsilon _{Q} = \frac{|Q_{\textrm{ELM}}-Q_{\textrm{3D}}|}{Q_{\textrm{3D}}}, \end{aligned}$$

where, $Q_{\textrm{ELM}}$ is the flow rate calculated by the ELM and $Q_{\textrm{3D}}$ is the flow rate calculated by the 3D FEM simulations. This error is calculated for all 49 geometries and plotted as a function of the maximum gradient in gap size in Fig. 30. Therefore, this figure shows the contour plot based on 49 simulation results.

Figure 30 can be used to get an indication of the error in the flow rate prediction obtained from the ELM for a certain spatial variation in gap size.

## Appendix 4: Transient CFD simulations of the diastolic phase

To validate the steady-state assumption used in the CFD model presented in Sect. 4.2, transient simulations of the diastolic phase are performed using the fluid volume geometries of the ‘small nodules’ and ‘big nodules’ artificial case (both including multiple calcification nodules).

### Numerical method

The numerical method of the transient simulations is very similar to the steady-state simulations. Similar approaches for transient simulations can be found in literature (Prisco et al. 2022; Mao et al. 2018). In analogy to the ELM, blood is modeled as a homogeneous, incompressible Newtonian fluid with dynamic viscosity $\mu = 0.0035\;\text {Pa}\cdot \text {s}$ (Nader et al. 2019), and density $\rho = 1060\;\text {kg}/\textrm{m}^{3}$ (Luraghi et al. 2019). To calculate the flow, the Reynolds-averaged Navier–Stokes (RANS) equations are solved, considering the $\kappa$-$\omega$ SST turbulence model. The $\kappa$-$\omega$ SST model was selected due to the presence of free leakage flow jet stream near the tissue below the TAVR. The time step $\Delta t= 0.0001$ s is used in the transient simulations. The boundary conditions for the transient simulations are applied to the same domain as in the steady-state simulations, presented in Fig. 8b. At the inlet a transient pressure $p_{\textrm{in}}(t)$ is applied, based on the measured diastolic pressure difference in the in vitro experiments. At the outlet, a pressure $p_{\textrm{out}}=0$ mmHg is applied. The applied pressure $p_{\textrm{in}}(t)$ is plotted in Fig. 31. The time-averaged pressure difference during the simulated diastole equals 100 mmHg (Feher 2012), so that the results can be compared to the steady-state simulations presented in Table 2. Furthermore, a no-slip condition is applied to all other walls.

### Results

The leakage flow rate *Q* is determined by multiplying the area-averaged velocity perpendicular to the outlet by the outlet area. The time dependent regurgitant flow rate is compared to the steady-state result and is plotted in Fig. 32.

The time-averaged regurgitant flow rate obtained from the transient simulations is 62.14 ml/s, whereas a regurgitant flow rate of 63 ml/s was obtained from the steady-state simulations. A transient CFD simulation was also performed for the ‘big nodules’ artificial case. The obtained flow rate is again compared to steady-state CFD simulations and the ELM and is plotted together with the time-averaged flow rate in Fig. 33. Here, the time-averaged flow rate obtained from the transient simulations is 120.2 ml/s, whereas the steady-state CFD simulations resulted in a flow rate of 120 ml/s. Since the relative difference between the two CFD approaches is $<2\%$ for the ‘small nodules’ case and $<1\%$ for the ‘big nodules’ case, the steady-state assumption, used in the CFD model presented in Sect. 4.2, is considered to be justified

## Footnotes

1 MATLAB R2021a: MathWorks, Natick, Massachusetts USA.

2 The synthetic patient geometries are provided by the German institute of computer-assisted cardiovascular medicine, Charité, as part of the European Union’s Horizon 2020 research and innovation program under grant agreement No. 101017578 (SIMCor).

3 HyperMesh 2022: Altair Engineering, Troy, Michigan USA.

4 Portico TAVR: Abbott Cardiovascular, North Chicago, Illinois.

5 https://www.blender.org/

6 OpenCFD Ltd, Delaware, USA.

7 Ansys Fluent 18.0: Ansys Inc., Canonsburg, Pennsylvania USA.
